# Why we need dedicated insect microphones - A comparison between measurement and MEMS microphone arrays highlights gap in available hardware

**DOI:** 10.1371/journal.pone.0350946

**Published:** 2026-07-08

**Authors:** Jelto Branding, Dieter von Hörsten, Elias Böckmann, Jens Karl Wegener, Eberhard Hartung

**Affiliations:** 1 Julius Kühn Institute (JKI), Institute for Application Techniques in Plant Protection, Messeweg 11/12, Braunschweig, Germany; 2 Julius Kühn Institute (JKI), Institute for Plant Protection in Horticulture and Urban Green, Messeweg 11/12, Braunschweig, Germany; 3 Christian-Albrechts-Universität zu Kiel, Institute of Agricultural Process Engineering, Max-Eyth-Str. 6, Kiel, Germany; Teikyo University Hospital Mizonokuchi, JAPAN

## Abstract

Acoustic insect recognition could potentially become a key element of insect sensing devices for various applications in pest management and ecological surveying. However, research in this field has yet to bridge the gap between successful laboratory studies and real-world applications. This study reports on extensive experiments and simulations carried out that aim to quantify the influence of different microphone qualities on the prospects of acoustic insect recognition systems that use them. The first microphone used is a four-channel, high-end, low-noise measurement microphone array that represents the cutting edge of acoustic recording capabilities. The second microphone is a low-budget six-channel micro-electro-mechanical system (MEMS) microphone array, initially designed for home voice assistant applications. The study clearly shows that higher-sensitivity microphones will allow for the recording and recognition of much smaller and quieter insects. However, even the simple MEMS microphones can be used to successfully recognise some insects, even in simulated real-world noisy conditions.

## Introduction

Acoustic recognition systems could play a key role in facilitating digital insect detection [1,2]. Enabling digital systems to access the presence or absence of insects is much needed to promote the advancement of digital pest management systems [3] as well as digital biodiversity monitoring solutions [4].

Thus far, most research on this topic should be regarded as being stuck in the proof of concept phase. While some advances towards commercial use have been made in acoustic storage pest detection [5], most other fields show promising proofs of concept, but are still awaiting large-scale practical application [6–8].

A key step on the path from good laboratory results using expensive laboratory equipment towards the practical implementation of commercially available devices, is the development of purpose-built, low-cost microphones. Because of their abundant use in modern smart devices, micro-electro-mechanical system (MEMS) microphones currently have minimal production costs, making them ideal for use in commercial acoustic insect detection devices. However, most of these microphones are designed specifically for human voice applications, and their frequency response has been optimised for this task. In practice, this means that most available MEMS microphones will have sub-optimal sensitivity to the low-frequency sounds that are most relevant for most insect sound recognition tasks [9] but are of no benefit in human voice recognition systems.

Therefore, the next step towards commercially successful acoustic insect detection devices for a broader range of insects should be the development of purpose-built, low-frequency optimised, low-cost MEMS microphones. The aim of this study is to advance this process by estimating the influence of microphone quality on the prospects of acoustic insect detection systems. By providing a benchmark for what performance can be expected from what system, this work hopes to provide a starting point for the development of such purpose-built insect microphones.

This study involves a simulation approach. All insect sound recordings processed were made in an acoustic laboratory. Mixing these with background sound recordings allows real-world sound conditions to be simulated. Ultimately, digital insect detection is a task that needs to be solved in all sorts of environments, ranging from indoor locations, such as warehouses and storage units, to outdoor applications in fields, plantations and woods. This study employs an intermediate level of difficulty, lying between the controlled conditions of indoor scenarios and the full unpredictability of outdoor recordings by simulating the acoustic background conditions in a greenhouse with open windows and focusing on insects common in European greenhouse environments.

The work focuses on array assemblies rather than single microphones. Previous work showed significant advantages of using multiple microphones simultaneously, not just for related fields [10], but also specifically for acoustic insect detection [11]. The reader interested in a discussion on the advantages of using microphone arrays over single microphones in the context of acoustic insect detection is referred to [11], a work directly preceding this study. Therefore this research aims to evaluate the influence of microphone quality on the performance of acoustic insect detection systems by comparing not just different microphones but two microphone arrays: the measurement microphone array (MM) and the ReSpeaker Core V2.0 (RS). Whereas the MM represents a high-end, high-cost research approach intended to represent the cutting edge of technical feasibility, the RS was selected to resemble a system intended for commercial use in terms of pricing and performance.

The two systems are compared in two steps. The systems were used mostly in parallel to create two respective insect sound datasets composed of recordings of nine different beneficial and pest species common in European greenhouses. Comparing the size and analysing the content of these two datasets provides initial insight into the effects of microphone quality on the functionality and prospects of acoustic insect detection systems.

The two datasets were then used to train and compare two identical deep learning models in an environmental sound simulation. The model structure has been shown to perform well for this task [11], and the special training procedure used here represents a further advancement in work preceding this model in the environmental sound simulation. This comparison gives some insight into the level of microphone sensitivity that is of most benefit to these insect detection models.

## Material: Acoustic recordings

Working with insect sounds is a challenging acoustic recording task. This section describes the equipment and experimental setup used to generate the two sound datasets for this study. A more detailed elaboration of the method development behind this process was published along with the InsectSound1000 [12] dataset that has been partially used here.

### Recording hardware setup

**Fig 1:**
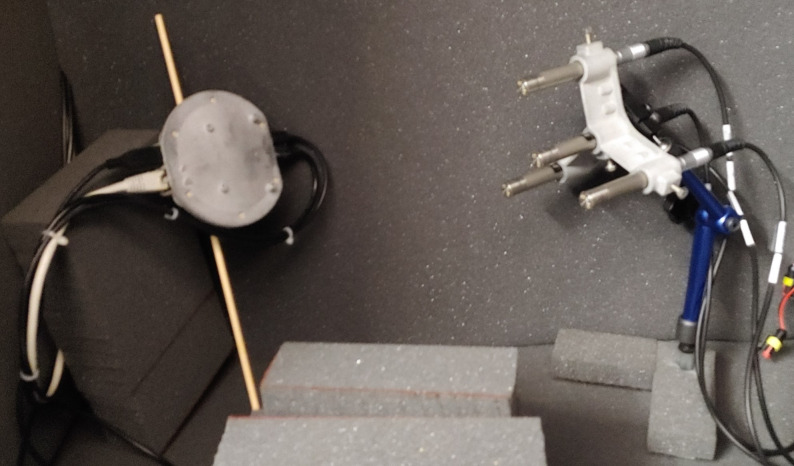
Recording hardware used. The low-noise measurement microphone array assembly (right) and the ReSpeaker Core v2.0 MEMS microphone array in its custom-made housing (left), pictured inside the anechoic box.

Previous work has shown that the use of multiple microphones, assembled to form an array, can provide significant advantages for the task of acoustic insect detection in noisy environments [11]. Therefore, this study investigates microphone array assemblies rather than individual microphones. The two arrays chosen for use in this study are described below, compared in Table 1 and shown in Fig 1.

**Table 1:** Table comparing the properties of the two microphone arrays and their microphones as given in their data sheets [13,14].

	Measurement Microphone Array (MM)	ReSpeaker Core v2.0 (RS)
Microphone Type	Externally polarised condenser microphone	MEMS
Microphones used	Brüel & Kjaer Type 4955	Knowles SPU0414HR5H-SB
Number of microphones	4	6
Microphone sensitivity [dB V Pa^-1^]	2.3	−22
Microphone sensitivity [mV Pa^-1^]	1300	79
Microphone noise floor [dB (A)]	< 6.5	35
Microphone SNR [dB (A)]	87.5	59
Array geometry	Star-like shape, one in the middle, three on a radius of 55 mm around the middle one	Circle of 92.6 mm

#### 2.1.1. Measurement Microphone Array (MM).

**Fig 2:**
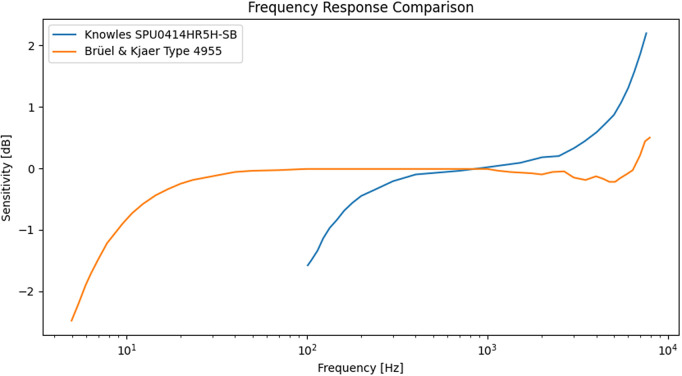
Microphone frequency response comparison. Plot comparing typical free-field frequency responses of a Brüel & Kjaer type 4955 low-noise measurement microphone and a Knowles SPU0414HR5H MEMS microphone. The Brüel & Kjaer type 4955 is used in the measurement microphone array and the Knowles SPU0414HR5H is used in the ReSpeaker Core v2.0. As is relevant to this study, the frequency response up to a frequency of 8kHz is plotted. The data for this plot was generated by digitalising frequency response plots given in the respective microphone data sheets [13,14].

The goal of the MM device in this study was to highlight the cutting edge of technical feasibility in order to obtain good data on the limits of acoustic insect detection. To exclude the possibility of a microphone of higher quality improving on the results, the aim was to select the best microphones available. When comparing microphones, a high sensitivity was prioritised over a low self-noise level [12]. Fig 2 shows a comparison of the frequency responses of the two microphone types used in the two array systems.

The MM is an array consisting of four low-noise, Brüel & Kjaer type 4955 measurement microphones. They were assembled to form an array using a self-designed 3D-printed fixture made from PA-12 (CAD file supplied as supplementary material S1 CAD File). The fixture places the microphones in a star-like shape, seen in Fig 1. The detailed properties of this array assembly are listed in Table 1 in the MM column. The reader interested in further details regarding the Brüel & Kjaer type 4955 measurement microphones shall be referred to the data sheet [13].

The MM was powered by a Brüel & Kjaer Nexus 2690 Conditioning Amplifier. Using the Nexus, the four signals were high-pass filtered at 20 Hz, low-pass filtered at 10 kHz, and amplified by 20 dB. The analogue signal was then fed to an A/D converter made by Roga Instruments called DAQ4. The gain function on the DAQ4 was disabled, AC-coupled mode was enabled and a digital high-pass filter with 0.3 Hz was activated. All recordings were digitalised using a sample rate of 48 kHz using the software DasyLab 2022 and saved as single-precision (32-bit, floating-point) TDMS-files, each 14:13 min long.

#### 2.1.2. MEMS Microphone Array (RS).

The RS device in this study was selected to represent the properties and capabilities of low-cost hardware that is priced so that it might find its way into commercial use for the task of acoustic insect detection with potential use cases in pest monitoring or large-scale biodiversity monitoring.

A review of available products and their usability for this research revealed that arrays made for use in so-called maker projects were the best fit. Many of these devices employ multiple high-quality MEMS microphones and can be bought as a small printed circuit board (PCB), integrating the microphones in a small computer board. Based on a review of the MEMS microphones used in various available products, and comparing mostly the number and sensitivity of the different microphones used, the ReSpeaker Core v2.0 from Seeed was selected. This product is a small PCB containing six MEMS microphones arranged in a circle along with a mini computer. The detailed properties of this array system are listed in Table 1 in the right column. The reader interested in further details regarding the MEMS microphones this system uses or the technical properties of the RS shall be referred to the respective data sheets [14,15].

A housing was designed around the raw PCB of the RS to allow its use under greenhouse conditions. The design goal was to shield the device from environmental influences but not to influence the acoustic recording capabilities. The resulting design is a two-part housing made from 3D-printed PA-12 (CAD-files are supplied as supplementary material S2 CAD File and S3 CAD File). On the non-microphone side, an 50 mm by 40 mm by 5 mm aluminium block acts as a cooling body. This is attached to the hot parts of the PCB via a thermal pad. The microphones are not covered by the housing. Instead, the microphones are covered with a 160 µm mesh to prevent small insects from entering the device. A thin 3D-printed silicon seal surrounds the microphones to seal the PCB against the housing and mesh (CAD file supplied as supplementary material S4 CAD File). The remaining gaps between the housing parts and around the various attached cables were sealed using silicon sealant. This design resulted in a housing (see Fig 1, left) that should prevent the ingression of dirt and insects into the sensitive electronics of the device while minimising any interference with the device’s recording capabilities.

The RS was set up using the headless version of the recommended Linux system. The recording properties of each of the six microphones in the RS can be influenced by two settings set in the “alsamixer” environment in its operating system: a programmable gain amplifier (PGA) value and a volume value. For the recordings processed in this study, the PGA of every channel was set to 100, and the volume of every channel was set to 63. Recordings were made using the “arecord” standard Linux tool. In this study, “arecord” was called up through a Python program that set the sample rate to 48 000 Hz and the encoding to signed 32 bit little-endian. After a maximum file time of 15 min, a new file was saved to an attached USB flash drive.

### Acoustic lab environment: Anechoic box

An anechoic box was built to shield the recording environment from environmental noises. The design consisted of two wooden boxes lined with different-density acoustic foams. By suspending the smaller box from springs inside the bigger box, a double wall structure was obtained that provided good noise shielding for frequencies above 100 Hz. For more details on the development, design and testing of this anechoic box, please refer to [12].

A 32.5 cm×32.5 cm×77 cm insect-rearing cage made from all-mesh material was placed inside the box. The cage was set up to contain two small tomato plants. Two types of LED stripes placed on top of the cage provided light. The light setup was slightly changed for recordings of *Trialeurodes vaporariorum*. Because the LED lights strongly attracted these insects, the lights needed to be positioned on the side wall of the rearing cage at the height of the microphones to guide these insects closer to the microphones. Due to their very low-level flight sounds, the original recording setup with the lights placed on top of the cage was unsuccessful, as insects flying around the lights placed on top of the cage were too far away from the microphones to be recorded. The recordings were conducted overnight to minimise the influence of noise from people working in the building. The setup of the cage and lights is described in further detail in [11] and [12].

The MM was mounted to a fixture and placed outside the cage, directly facing the mesh, very close to, but not touching the mesh. The RS was placed inside the cage by attaching the housing to a wooden stick placed inside the potting soil. Data and power supply cables attached to the RS were routed out of the cage through a mesh sleeve attached to one of the cage walls. Both arrays were placed approximately 30 cm above the box and insect cage floor.

### Insect recordings

The purpose of the original project was to build a system applicable to the context of protected horticulture. Therefore, all insects recorded are likely to be found in a European greenhouse. Further, the goal was to portray a range of different sound levels. Table 2 gives an overview of the insects selected for this study, their body length and average sound pressure level (SPL) from the recordings. The range of insects selected extends from small flies to bigger bugs and covers some beneficial as well as pest insects. Expanding on previous work [11], this study includes insects that were difficult to record and yielded a very low sound sample number, regardless of extensive recording efforts. Examples of this are small pest insects such as *Myzus persicae, Trialeurodes vaporariorum* and *Tuta absoluta*. The MM data used in this study is a subset of the InsectSound1000 dataset that was published in [12]. The RS data used in this study is unique to this study and a subset of the InsectSound1000-MEMS dataset available at [16]. Fig S1 Fig allows for a visual comparison between the different insect sounds and the effect the different recording hardware has on the actual data recorded. The Fig contrasts selected spectrograms of the same insect species recorded with the two different microphone arrays. As this study did only involve non-vertebrate insects, no permits were required under German law for the recordings made in this study.

**Table 2:** Overview of insect species investigated.

Insect species	Adult body length [mm]	Mean sample SPL in dataset [dB]
*Aphidoletes aphidimyza*	1–3	10.7
*Bombus terrestris*	11–17	25.9
*Bradysia difformis*	3–4	9.3
*Coccinella septempunctata*	5–9	12
*Episyrphus balteatus*	7–12	18.5
*Myzus persicae*	2	9.6
*Rhaphigaster nebulosa*	14–16	11.8
*Trialeurodes vaporariorum*	1–2	9.7
*Tuta absoluta*	6–7	13.4

As derived in [12], the experimental setup, which physically ensures all sounds recorded in one session must come from one species, represents the only way to generate labelled insect sound recordings. By placing only insects of one species inside the anechoic box at a time, all sounds recorded in one session should be from this one insect species.

For every recording, only one cage containing three to 50 insects of one species was placed inside the anechoic box. Bigger, louder insects were recorded using only a few individuals, while smaller insects were recorded in higher numbers to ensure that three to four nights of recording time would yield roughly the same amount of insect sound events in the recordings.

Unfortunately, not all recordings used in this study were recorded simultaneously using both microphone arrays. Varying technical problems with both microphone arrays meant that on some nights valid data from only the RS could be saved, while on other nights, only the MM recordings produced valid data. Out of a total of 48 recording nights processed in this study, three produced valid data for only one of microphone arrays. In an effort to maximise the available data to train each of the two models, it was decided against discarding the data recorded on these semi-successful recording nights. In total, 742 h of MM recordings and 709 h of RS recordings were collected and used for further data processing.

### Environmental sound recordings

Background sounds were recorded in a greenhouse and used to simulate noisy conditions by mixing the insect sound recordings from the acoustic lab with these background sounds from the greenhouse. The greenhouse was located at the facilities of the JKI in Braunschweig and used for research purposes, therefore no permits were required for the recordings made in this study. Six different locations inside the greenhouse were simultaneously recorded using both microphone arrays, in the same way as for the laboratory recordings. At every recording location, two different orientations of the microphone arrays were recorded for 5 min. The process for generating this environmental sound dataset is described in detail in [11]. The dataset containing the simultaneous background noise recordings of both microphone arrays is available at [17].

## Method: Data processing

The model architecture and training data pipeline used to train the two models compared in this study are presented below. The approach represents an attempt to improve the results of training neural beamforming (NBF) WaveNet models in the environmental sound simulation originally proposed in [11]. S1 Table summaries the differences in data processing between [11] and this study.

### Model architecture

This study processed multichannel data from both the MM and the RS. It is fundamental to this comparison that the deep learning models utilised here can exploit the benefits of this multichannel data. Deep learning classification models directly processing raw audio data have been proposed for such applications [18]. Previous work has shown the benefits of such multichannel raw audio data processing models for insect sound recognition, especially when dealing with strong background noises [11]. This study uses the same model as that proposed in [11] to compare the potential of the two microphone array setups. To process the RS data, only the input size was modified from four to six channels. This raw audio data processing acoustic detection model will be referred to as the NBF WaveNet.

As the name suggests, this model is made up of two components. The NBF layer, for neural beamforming is designed to learn spatial filtering by mimicking the calculations of a filter and sum beamforming algorithm. In classic signal processing, such beamforming algorithms are used to extract directional information from multichannel sound signals [19] as a part of signal preprocessing. In this study, the data was not explicitly preprocessed using such spatial filtering. Instead, the NBF layer is part of the deep learning model and trained to perform similar calculations implicitly.

This second part of the model is a WaveNet classifier used to process and classify the raw, now single-channel, prefiltered signals generated by the NBF layer. Originally introduced as a generative model for music and speech synthesis [20], the WaveNet architecture has since been shown to be a powerful sound classification model [21–23]. A more detailed description of the implementation, based on [24] and [23] is given in [11]. The Jupyter Notebook containing the full code used in this study is available at [25].

### SPL-Weighted loss

Previous work has shown that the NBF WaveNet model used in this study is a significant improvement upon a naive single-channel spectrogram-based model as it effectively utilises spatial information encoded in the multichannel recordings. However, the model also turned out to be difficult and unstable to train, as only two out of five training attempts converged in all of the training steps in the environmental sound simulation in a previous study [11].

It is well understood that the first layers of a deep learning model are the most difficult to train. This is because rounding errors will degrade the training signal (gradient) when backpropagating through the model from the last to the first layer during training, famously called the vanishing gradient problem [26]. In the case of the NBF WaveNet model, this very first layer, however, is the NBF layer, which is of great importance to the overall model performance, as it is the only part of the network that can learn spatial filtering capabilities. Combining the difficulty of training this first layer with its importance makes for difficult and unstable model training.

In addition, the dataset of insect sounds in the environmental sound simulation must be ranked as very challenging, or even partially unsolvable. When simulating full-scale environmental sounds, the SNR is much smaller than in related tasks [27] and can sometimes drop below −30 dB. Previous work concluded that this dataset of insect sounds contained so many very quiet, and therefore very difficult to classify, samples, it seemed to hinder model convergence during training. This led to a sub-human performance even on louder, easy-to-classify samples in some models trained and tested in simulated greenhouse conditions [11].

In an attempt to stabilise training in the environmental sound simulation, this study proposes the use of a difficulty-weighted loss function for training in simulated noisy conditions. Its idea is to mimic the approach of a human learner tasked with solving a partially unsolvable problem. By simply multiplying the commonly used categorical cross-entropy loss value with a measure of classification difficulty, getting the easy samples right is emphasised during training.

Quantifying classification difficulty for individual samples is a very much non-trivial task in most fields (e.g., image recognition). For the task of audio classification in the presence of noise, however, the SNR of a given sample provides a simple and reasonable estimate of classification difficulty. This is as long as the frequency content of both the noise and the signal dataset do not vary too much between samples. In theory, if noise and signal sounds are of purely different frequencies, they can be distinguished no matter the SNR. In practice, the sound samples used to represent the environmental sounds in this study contain signals mostly described by a $\frac{1}{f}$ distribution (pink noise). The insect sound samples, while specific to each of the classes in detail, can collectively be assumed to have a relatively similar frequency distribution.

Furthermore, given that the environmental noise simulated is roughly the same level for every sample, calculating just the insect sample SPL alone is sufficient to provide a good approximation of classification difficulty in this study. By writing the SPL of every sample to its filename and simply reading these values while building the dataset, the proposed loss function provides close to no computational overhead during training. The SPL was calculated in the logarithmic unit dB to provide values in a reasonable value range. This leads to the following formulation of the proposed loss function in equation 1:


$$\begin{array}{c} Loss_{SPL-weighted}=CCE\times SPL\;[dB] \end{array}$$ (1)

where *CCE* denotes the commonly used categorical cross-entropy loss function. Preliminary experiments showed that while final results at the end of all training steps in the environmental sound simulation did not vary significantly, training in the early steps was accelerated, and convergence was stabilised when using the SPL-weighted loss function as soon as the simulated noise amplitude is greater than zero. Contrary to a priori excluding the quieter, more difficult samples from training, training on the full dataset with the proposed loss function allows the training process to determine what is possible or impossible to hear.

### Model training

Training a deep learning model involves making several choices which determine the success of the undertaking. The parameters of model training that lead to the results presented in this study, as well as their choice, are described briefly below.

#### 3.3.1. General training pipeline and hyperparameter choice.

Training and evaluating the models in the environmental sound simulation adds some extra steps and complexity to the data pipeline. Additionally, the big difference in the number of samples per class, especially for the RS data, made balancing the dataset necessary. This was done by over- and under-sampling the available samples of every class to a target number of training samples. Balancing the datasets was implemented using a deterministic resampling function, meaning that calling the function twice with the same target number of samples will yield the same list of samples.

Using this function, both models were trained with 2350 training samples per class, which is the number of samples in the biggest class in the RS dataset, in an effort to minimise the effect of the different dataset sizes on the comparison of the two models. The number of validation samples per class was capped at 1000, meaning the validation dataset was not upsampled but downsampled if the number of samples per class exceeded 1000 samples to minimise training time. The test dataset was not resampled at all.

Full details of the steps of the training data pipeline are provided in S1 Appendix. The training pipeline as well as the models were implemented using TensorFlow 2.10 [28]. Both models were trained using the Adam optimiser [29], set up with the default values implemented in TensorFlow. The training was automatically terminated using early stopping, with a patience of 20 epochs monitoring the validation loss. Both models were trained using a batch size of 64 on a machine equipped with a NVIDIA GeForce RTX 3090.

#### 3.3.2. Data augmentation.

The correct and plentiful use of data augmentation has been shown to play a vital role in improving deep learning results. By deliberately randomly varying all parameters of the training data, which are a priori known to not correlate with the classes, one can ensure these parameters will not contribute to the decisions of the model [30].

This study also uses a few data augmentation operations applied to the insect sound data. The augmentations listed in S2 Table were applied at random or within a random value range. A more detailed discussion explaining the choice of augmentation techniques and their value range on this particular type of sound classification problem can be found in [11].

#### 3.3.3. Exposure to environmental noise sounds during training.

The augmentation methods described above are usually applied only to the training data. Additionally, this study employed an environmental sound simulation proposed in [11]. This simulation process mixes not only the training but also the validation and test data for the two microphone arrays, with recordings from the greenhouse made with the corresponding array. Neither model converged when exposed to insect sounds mixed with full-scale environmental sounds during training from scratch. However, training the same models on the clean insect recordings from the acoustic lab was unproblematic. As a solution, a fine-tuning approach was applied. By training the models on clean data and then continuing the training with gradually increasingly noisy data, model convergence was facilitated. Specifically, the two models pre-trained on the clean data were fine-tuned first on data mixed with environmental sounds at 1% of their original amplitude by multiplying the environmental sound signal with 0.01, and then adding up the insect and environmental sound signals. The fine-tuned models were then further fine-tuned on data mixed with environmental sounds at 10% of their original amplitude, and then again on data mixed with full-scale environmental sounds signals (100%).

#### 3.3.4. Learning rate choice.

When exploring similar training approaches using the environmental noise simulation in preliminary experiments and previous work, a strong sensitivity of the NBF WaveNet model employed to learning rate choice became apparent. Previous work found that increasing the learning rate from an initial $9\times 10^{-5}$ for training without noise to $5\times 10^{-4}$ when restarting training with mixed-in noise was the only learning rate tested that achieved successful training of the NBF layer filters. These models showed an improvement of roughly 20% in classification accuracy, compared to NBF WaveNet models that could not effectively use their NBF layer [11].

For this reason, an extra careful approach was taken when choosing learning rates for these experiments. To ensure learning rate was appropriate and avoid extensive grid search trials, the learning rate test proposed in [31] was performed for both models before every training step in the environmental sound simulation.

To further mitigate the influence of learning rate choice on the results, models were trained with cyclical learning rates as proposed in [31]. Base and maximum learning rates were chosen based on the results of the learning rate test for each model individually by manually evaluating the progression of the training loss during the test. These are listed in S3 Table. The cyclical learning rate was applied using a decreasing cyclical learning rate scheme (called “exp_range”) with a gamma value set to 0.99994 and a cycle length of two training steps.

### Model evaluation

Model testing was also done in the environmental sound simulation in the same way as for training and validation. Test data was generated by mixing sounds from separated insect sound test sets and environmental sound test sets. Because the environmental sound samples are chosen at random from the environmental sound test set, the evaluation metrics fluctuate slightly. Depending on whether a quiet insect sample is mixed up with a loud or a quiet environmental sound sample, it will be either easier or more difficult to classify. Therefore, all evaluations involving data containing simulated background sounds were repeated 10 times to account for these fluctuations. The results presented here are the mean values of these ten repetitions.

Because the models are initialised with random values at the beginning of training, the results vary slightly for each training repetition. Each model was trained five times on clean data. The best three models, or however many converged during this first step of training, were then subsequently fine-tuned and refine-tuned at increasing noise amplitudes following the process described in Model Training.

### Data preparation

The data pipeline feeding the data to the deep learning models involved many steps, from recording the raw audio data to model training and testing. Most of these steps, however, are identical to a previous study based on a subset of the MM recordings [11]. Therefore, only a summary of the data pipeline is provided below.

#### 3.5.1. Sound sample extraction.

To create the two datasets from the insect sound recordings made in the anechoic box using the two different microphone arrays, the actual sound events needed to be extracted from the mostly silent recordings. In line with the preceding work, this study processed 2500 ms long samples, downsampled to 16 kHz. As the MM data used in this study is a subset of the InsectSound1000 dataset, a detailed description of the sample extraction process has already been published in [12] as well as a Jupyter Notebook implementation of the method.

In short, the segmentation processed is based on the idea of activity detection on a prefiltered signal through thresholding the signal energy contained within a sliding window moving over the recording (loosely based on [32]). The sections containing acoustic activity are then grouped into non-overlapping samples of equal length. Only two adjustments were made when processing the RS data, to account for the lower microphone sensitivity and the resulting different noise levels in the recordings:

- Adjusting the threshold for window energy from 1.6 to 1.4 times the mean of all the window energies found in the recording.
- Adjusting the minimum activity segment length from 1 s to 0.25 s, meaning that activity segments shorter than this, without any previous or following activity within a 2500 ms range, were discarded as noise.

These two datasets were then each split into training, validation and test data. Preliminary experiments indicated that some powerful models could learn not only to distinguish the different insect species, but also the different recording days on which one insect species was recorded [12]. To prevent data leakage from training to validation and test set during model training, the datasets were split, separating the different recording dates. This means that the models are validated and tested with sounds recorded exclusively on different days than those used for training. Unfortunately, this means the training set for some insect species was smaller than the common 60% of the dataset, while the validation and test set might be bigger than the common 20%. This occurred if, for example, the three recording nights on which one species was recorded had to be used each as a train, validation and test set.

To create a corresponding set of noise sound files, the environmental sound recordings made in the greenhouse, as described in section Environmental Sound Recordings, were also split into non-overlapping 2500 ms segments and saved as 16 kHz files. To ensure both datasets were completely identical, the RS and MM raw sound files were cut into completely simultaneous slices, based on time stamps manually measured in the recordings of both arrays. In this way, a dataset of 1495 environmental sound samples was created for each of the microphone arrays. These two datasets were then also split into files used for training, validation and testing using a 60-20-20 split. The two datasets were split simultaneously to ensure comparability of test results. This means that the files contained in the RS background sound test dataset, for example, are recordings of the same moments in the greenhouse as the recordings in the MM test dataset.

#### 3.5.2. SPL calculation.

To employ the SPL-weighted loss function proposed in section Model Architecture, the SPL value in dB is calculated from the raw multichannel signals for both microphone arrays and added to the end of each insect sound sample filename during data preparation.

As the name suggests, the measurement chain of the MM consists of calibrated devices that allow the exact correlation of the values found in the recording file to the sound pressures in front of the microphone capsules. The detailed calculations are given in S2 Appendix.

In contrast, the RS lacks reliable calibration data. It is not possible to quantify the amount of gain applied to the signals by its internal processing. Therefore, an adequate approximation must be made to calculate SPL-like values for each of the insect sound samples in the RS dataset to process them in a model using the SPL-weighted loss function proposed in Model Architecture. Furthermore, this will allow for a more detailed comparison of the composition of the two datasets.

These SPL-like values were calculated by using the simultaneous background sound recordings made with both microphone arrays in the greenhouse to estimate an adjustment factor (details in S3 Appendix). Multiplying the RS signals with this adjustment factor converts the RS signals to the same value range as the MM signals. Based on these converted signals, an approximation of the sample SPL values can be calculated using the same steps as described for the MM SPL values.

This approximate calculation, however, is expected to contain a rather large error. This is because a single adjustment factor, as used here, cannot account for the different sensitivity to different frequencies. The high-quality measurement microphones in the MM possess an almost linear frequency response. This is not the case for the MEMS microphones built into the RS. Simultaneous test recordings with both microphones and 1 kHz test sounds in the acoustic laboratory suggest the resulting SPL values for the RS are likely overestimating the real SPL.

## Results

The results section first presents a comparison of the two different datasets investigated in this study, followed by the results of training two deep learning models on these datasets.

### Comparison of the two datasets

In total, 742 h of recordings were evaluated for the MM and 709 h for the RS. In general, the microphones of the MM generated recordings that allowed the extraction of more sound samples per hour. In total, 98 648 insect sound samples could be extracted from the MM recordings, whereas the RS recordings only yielded 9221 samples. On average, over all the insect classes, almost 11 times more sound samples could be extracted from the MM recordings than from the RS recordings. This must be explained, first, by the much greater microphone sensitivity of the microphones in the MM (see 1). Greater microphone sensitivity means that sounds either quieter or further away from the microphones are recorded and subsequently can be extracted. Second, the MM has an exceptionally low self-noise level. Lower self-noise in the measurement chain means that quieter or farther away sounds, if recorded, can be distinguished from the background noise of the recordings. As a big portion of the insect sounds is of very low intensity, this second threshold leads to a much worse performance of the relatively noisy RS compared to the MM.

**Fig 3:**
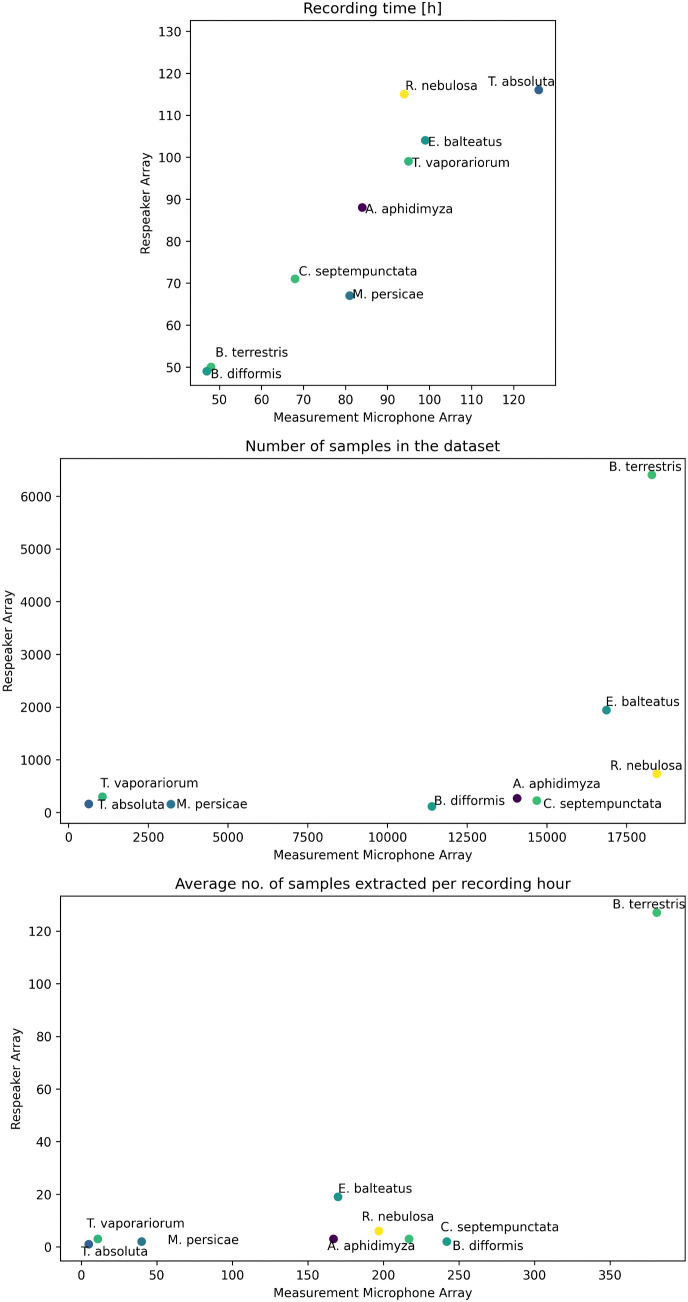
Scatter plots comparing the properties of the RS and MM dataset. The plots compare the recording time, the number of samples and the average number of samples extracted per recording hour.

Fig 3 shows three scatter plots detailing the numbers of the two datasets that form the basis of the insect detection system comparison. The first plot compares the number of recording hours evaluated for both microphone arrays. The second plot shows the number of sound samples that could be extracted from these recording hours for every insect class in the two datasets. The third plot compares the average number of samples that could be extracted per recording hour for the two microphone array setups to illustrate the varying difficulties in generating the datasets.

Unfortunately, not all recordings were done fully simultaneously due to various technical problems, as described in section Insect Recordings. This is why the number of recording hours used differs slightly for the two microphone array setups. This can be observed in the recording time plot of Fig 3. The data points of almost all insect species lie on the diagonal between the x- and y-axis, but the dataset of the RS relies on a few more recording hours for the *Rhaphigaster nebulosa* and on a few less for the *M. persicae* and *T. absoluta* compared to the MM.

By dividing the data from the second plot of Fig 3 (number of samples in the dataset) by the data from the first plot of Fig 3 (recording time), one can calculate the average number of samples extracted per recording hour for each insect species. This is depicted in the third plot of Fig 3. This value represents a sort of insect recording yield rate and gives an intuitive understanding of how difficult it is to generate a reasonable size dataset for each of the different insect species with either one of the two different microphone arrays used.

When evaluating this third plot of Fig 3, *Bombus terrestris* clearly appears to be the easiest to record with both microphone setups. Interestingly, the order of many other insects differs in this plot between the two recording setups compared. For example, *Episyrphus balteatus* has the second highest yield rate for RS recordings but is outperformed by many other insect species, including *Bradysia difformis* and *Coccinella septempunctata*, in terms of yield rate in the MM recordings. This must be explained by a different composition of the two datasets. While the MM dataset contains a mix of quiet and loud sounds, the inferior properties of the RS lead to a dataset containing only relatively loud sounds. As the ratio of low-level to high-level sounds differs for every insect species, so does the order of recording yield rates for the two datasets.

Besides the obvious influence of the different insects’ sound levels, a difference in audible insect activity has a big influence on the recording yield rate that is depicted in the third plot of Fig 3. Extreme examples of this are *T. absoluta*, which has a relatively loud flight sound but hardly ever moves when kept inside a small cage with a healthy tomato plant. On the contrary, the flight sound of *Aphidoletes aphidimyza* is so quiet, it is inaudible to human ears. These small insects, however, were very active during the recordings made in this study.

**Fig 4:**
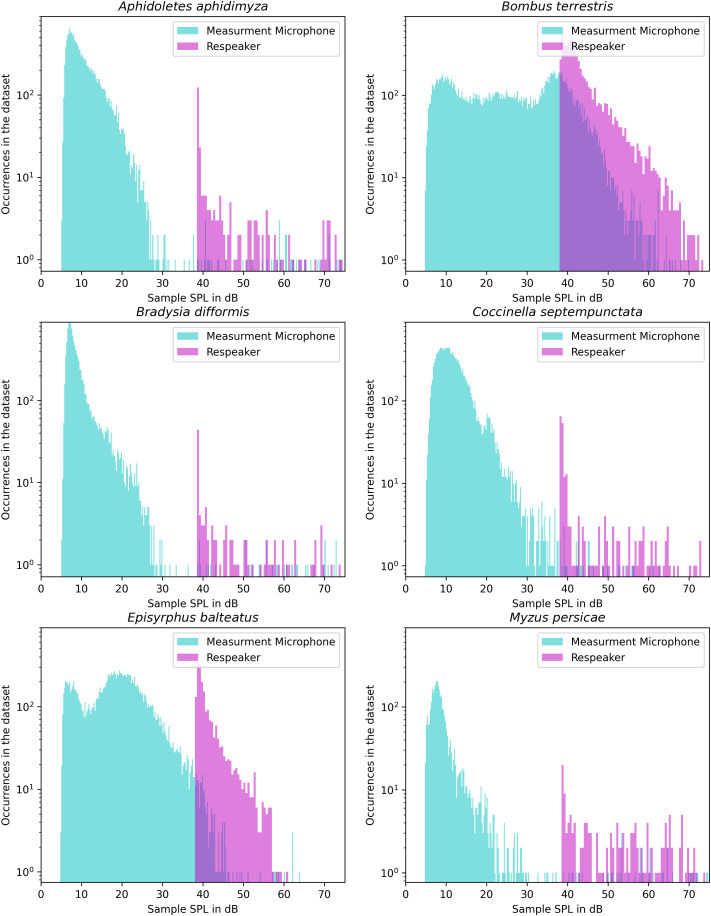
Histograms comparing the number of sound samples of a given sound pressure level (SPL) in the two datasets for the different insect species. SPL values for the RS dataset are estimated based on an approximation calculation, likely overestimating real values.

**Fig 5:**
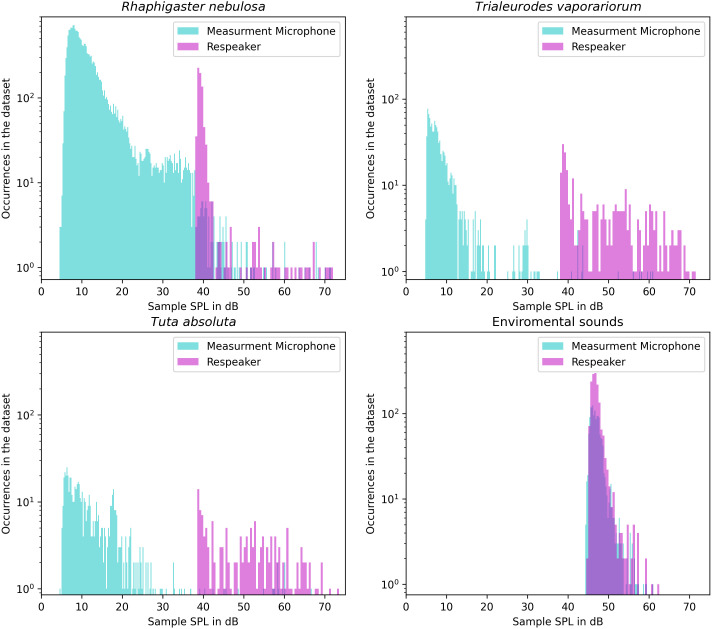
Histograms comparing the number of sound samples of a given sound pressure level (SPL) in the two datasets for the different insect species and the environmental sounds. Continuation of Fig 4. SPL values for the RS dataset are estimated based on an approximation calculation, likely overestimating real values.

The composition of the different datasets regarding the different sample SPL values is further detailed in Fig 4 and 5. These compare the SPL distribution of the sound samples of every insect species in the two datasets. At first glance, it becomes obvious that both datasets contain much more low SPL samples than high SPL samples. This is especially clear when observing the log-scale y-axes of these plots.

An analysis of the spectrograms representing samples of different SPL in the dataset showed that the higher-level sounds seem to clearly show flight sounds of the different insects, whereas lower-level sounds instead appear to be non-flight sounds originating from other types of insect motion (e.g., walking or folding and unfolding wings).

The comparison between the two microphone setups in Fig 4 and 5 clearly shows the ability of the MM setup to record and extract sounds of much lower SPL compared to the RS. While the quietest sound samples to be found for each of the displayed insects in Fig 4 and 5 in their MM datasets reach sound levels as low as 5 dB, none of the sounds in any of the RS datasets is below a SPL of 38 dB. This must be explained by the lower sensitivity and the greater self-noise of the RS system (see Table 1). These two properties combined lead to RS recordings that do not allow sounds below 38 dB to be distinguished from the background sounds and self-noise of the recordings.

However, while the general principle holds, it must be mentioned that the RS SPL values displayed in these plots in Fig 4 and 5 are calculated based on a rough estimate of a correlation factor (see section SPL Calculation) and must be interpreted with caution. Looking at the plot for *B. terrestris* and *E. balteatus*, it appears that the correlation factor used to estimate the gain in the RS system is likely leading to an overestimation of the real SPL values in the RS dataset. It appears unlikely that the RS recordings yielded more loud sound samples than the MM recordings, especially because the number of recording hours is identical (compare Fig 3), meaning these two insects were recorded fully parallel with both recording devices. From this reasoning, it seems the SPL values of the RS dataset are overestimated by up to 10 dB. Therefore, the real lower bound for insect sounds that can be recorded in such a way that allows for their extraction from the RS recordings could lie as low as 28 dB instead of the 38 dB as suggested by the plots in Fig 4 and 5.

Finally, the last plot in Fig 5 shows a comparison of the SPL distribution of the environmental sound samples recorded in the greenhouse with both microphone arrays. As was the objective of estimating the correlation factor (see section SPL Calculation), the two SPL distributions appear to be well aligned. Comparing their value ranges to the insect sound sample SPL distributions allows for an estimation of the SNR that the mixed recordings will yield during model training and testing in the environmental sound simulation.

**Fig 6:**
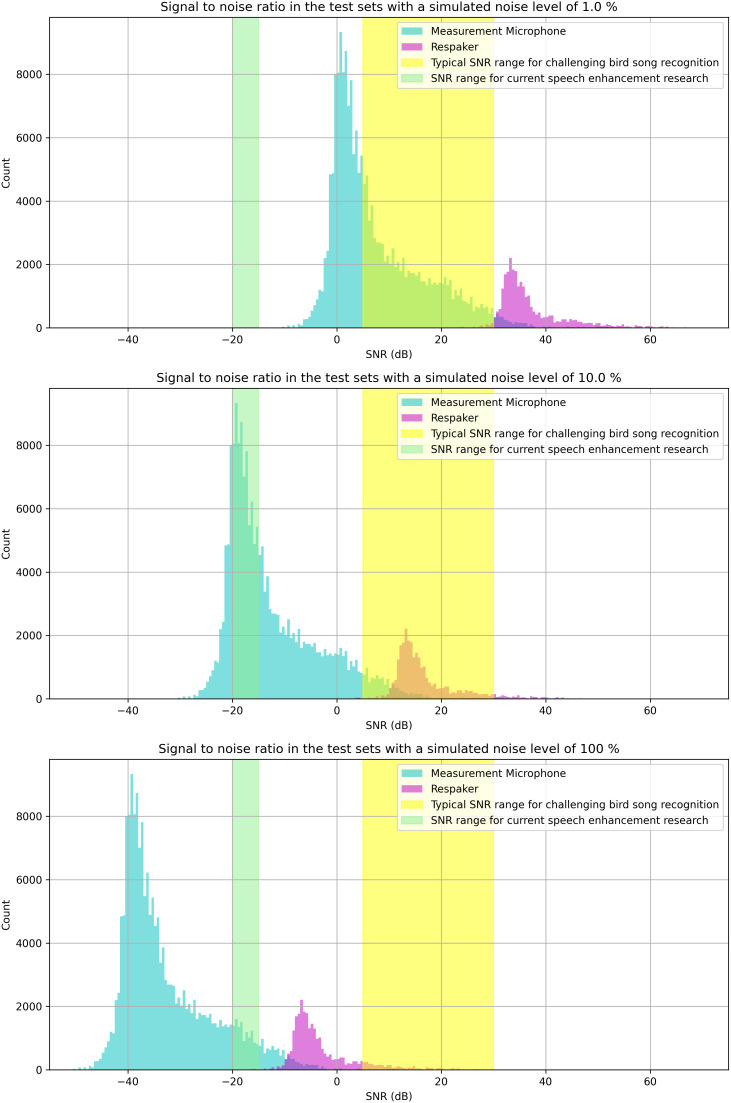
Histograms comparing the SNR distribution in tests generated from mixing insect and environmental noise sounds scaled to different noise levels for both microphone arrays. As described in section Model Evaluation, the test datasets are generated by mixing the insect sounds from the test set with randomly selected environmental noise sounds from the noise test set. The histogram was generated by running this randomised mixing process 10 times, as is done during model evaluation. The SNR was calculated by subtracting the SPL of the insect sound sample from the SPL of the environmental noise sound sample used for mixing. The SPL of the environmental noise sound samples was calculated in the same way as described in section SPL Calculation for the insect sounds. An operating range for bird song classification, that is considered challenging [33] and a SNR range for current speech enhancement research [34] are marked for comparison.

Fig 6 shows histograms comparing the SNR distribution in test datasets generated from mixing insect and environmental noise sounds scaled to the three different noise levels used during training and testing of the Deep Learning models presented in the following section. The figure also shows typical SNR ranges found in two related research areas, bird sound recognition [33] and human far-field speech enhancement [34], for comparison. The comparison clearly shows the extreme signal conditions that insect sound classification tasks are facing.

### Results of training two deep learning models on the different datasets

**Fig 7:**
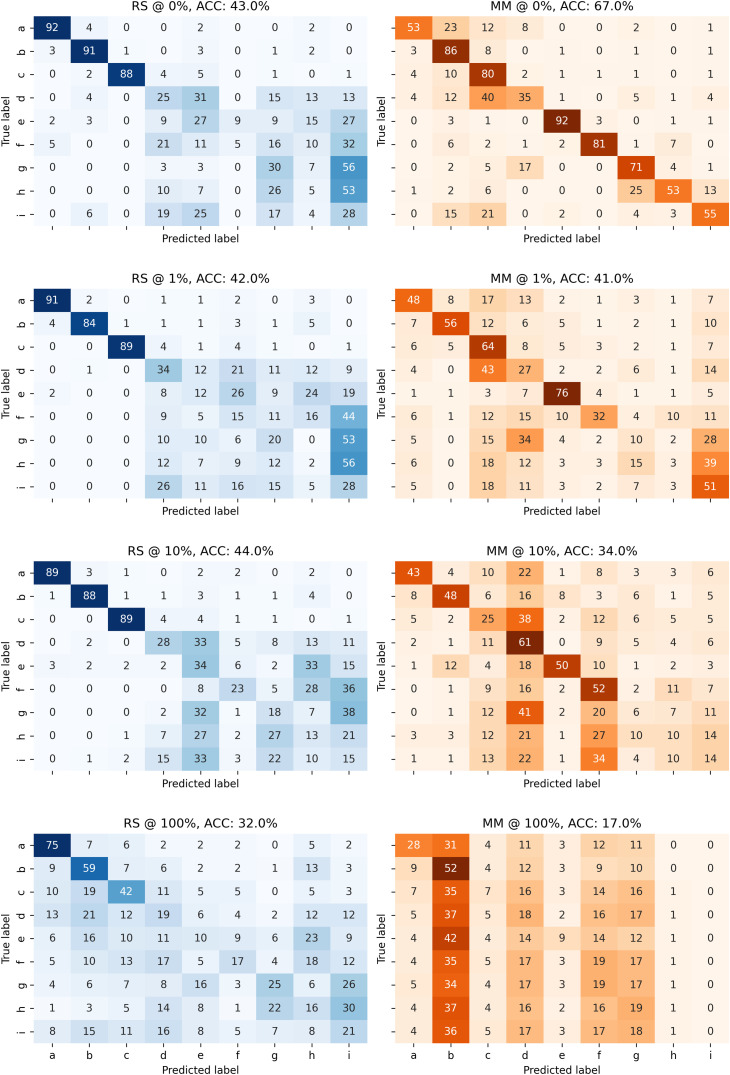
Confusion matrices comparing the best attempts of training an MM and RS model in the environmental sound simulation, showing relative values. Classes denoted as: a *Bombus terrestris*, b *Episyrphus balteatus*, c *Rhaphigaster nebulosa*, d *Coccinella septempunctata*, e *Aphidoletes aphidimyza*, f *Bradysia difformis*, g *Tuta absoluta*, h *Myzus persicae*, i *Trialeurodes vaporariorum*, in order of subjective classification difficulty.

**Fig 8:**
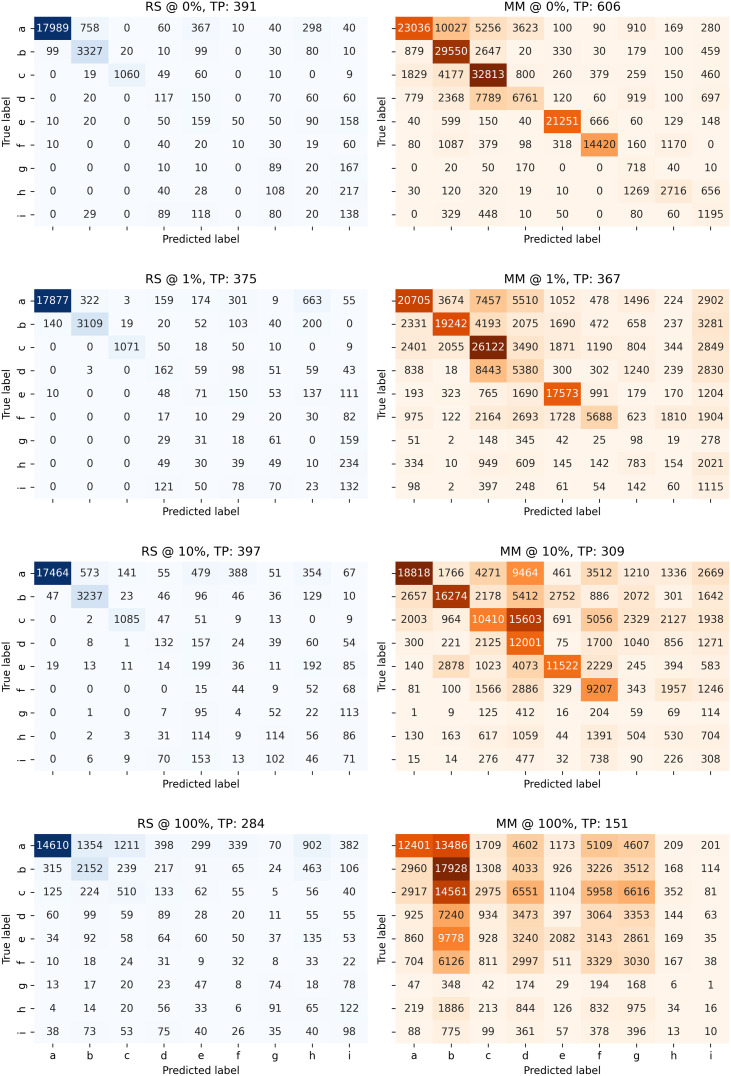
Confusion matrices comparing the best attempts of training an MM and RS model in the environmental sound simulation, showing absolute values. Classes denoted as: a *Bombus terrestris*, b *Episyrphus balteatus*, c *Rhaphigaster nebulosa*, d *Coccinella septempunctata*, e *Aphidoletes aphidimyza*, f *Bradysia difformis*, g *Tuta absoluta*, h *Myzus persicae*, i *Trialeurodes vaporariorum*, in order of subjective classification difficulty.

Fig 7 and Fig 8 show confusion matrices comparing the best results of the training attempts made on models using either the MM or RS data. Further, precision, recall and F1 scores of the models are given in S2 Fig. All models were trained to distinguish all nine of the species contained in this dataset. The results presented are the best runs out of five training attempts.

As to be expected, the results of training on the pure laboratory data, without the addition of any noise, show a much better result for the model trained on the MM data. The first confusion matrix displayed on the right (orange) in Fig 7 shows a clear diagonal, indicating that the model is correctly recognizing the insect sounds in the majority of the cases tested. The model trained on the RS data (Fig 7, left, blue,) on the other hand, only learned to recognise three out of the eight classes securely, albeit with higher confidence than the MM model.

This can be explained by the different volumes and composition of the two datasets, already discussed in section Comparison of the Two Datasets. The sparse data on some of the quieter insects and the greater imbalance between the classes in the RS dataset mean RS trained models struggle to pick up on the patterns of these underrepresented classes. Similarly, the different composition of the datasets means that the majority of the sounds contained in the RS dataset are louder and therefore easier to recognize when present in an adequate quantity to be learned by the model. The large proportion of very quiet sounds in the MM dataset means models trained on this data face a more difficult problem.

This becomes apparent when looking at the absolute numbers of test samples correctly classified displayed in Fig 8 instead of the relative values displayed in Fig 7. For example, the MM model was only able to correctly recognise 53% of all *B. terrestris* sounds without the presence of background noise, while the RS model was able to recognise 92% of them in the same conditions. However, in absolute numbers the MM model still correctly classified almost 500 sounds more than the RS model.

**Fig 9:**
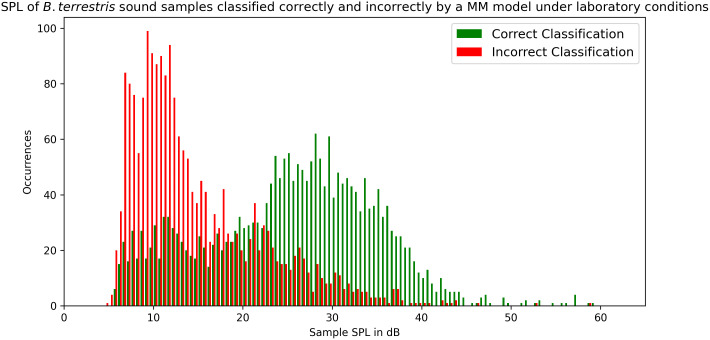
Histogram displaying the sound pressure level (SPL) of *Bombus terrestris* test samples that were correctly or incorrectly classified. Classification was done by the MM model trained and tested on pure laboratory recordings.

Fig 9 compares the SPL of samples that were classified correctly or incorrectly by the MM model for the class of *B. terrestris* in conditions without background noise. Combing these results with the comparison of the SPL distribution in the two datasets introduced in Fig 4 suggests that the different composition and volume of the two datasets mean that the quiet *B. terrestris* sounds the MM model is likely to misclassify are simply not present in the RS data.

When comparing the results of both models on the pure laboratory sounds with the results after retraining these models on data mixed with background sound recordings scaled down to 1% of their original amplitude, in Fig 8 one can observe a very different effect of the background sounds on the two models. While the RS model is barely affected by the presence of the low amplitude background sounds in the data, the MM model loses 26% of its average accuracy. Furthermore, the MM model starts losing the ability to recognise the sound of *T. absoluta* and *M. persicae* (“g” and “h” in Fig 8).

This different reaction of the two models to the introduction of even only very mild background noise must also be explained by the different composition of the two datasets. As the MM dataset contains mainly low-level sounds, these are easily masked when simulating the presence of background sounds, causing the model to struggle with their classification. The loud sounds contained in the RS dataset, on the other hand, do not get masked by the downscaled background sounds.

When continuing the training in the environmental noise simulation by training both models on sounds mixed with background sounds downscaled to 10% of their original amplitude, one even sees a slight improvement in the average accuracy of the RS model, as displayed in Fig 8. However, this is most likely just a numerical effect of the repeated training restarts, and not related to the changes in the data occurring in this step of training. The MM, on the other hand, loses the ability to recognise the sounds of *T. vaporariorum* (“i” in Fig 8).

Mixing the insect sounds with full-scale background sound recordings represents the best approximation of realistic sound conditions in the environmental sound simulation. When continuing the training of both models on data as such, one sees that both models are clearly affected by the presence of noise, as displayed at the bottom of Fig 7 and 8. The MM model loses the ability to recognise all but one class and starts guessing, as indicated by the column pattern in the confusion matrix. The RS model is also affected, as the full-scale background sounds are also louder than the majority of the sounds in the RS dataset. However, because of the different structure of the dataset, the model is still able to recognise most of the sounds in the three classes it was performing best on during the previous steps of training. Because the loud samples of these three classes make up a very big portion of the total dataset, their recognition remains stable. Therefore, this comparison surprisingly showed a model trained and tested on the lower quality and, for most classes, smaller RS dataset outperforming a model trained and tested on the MM dataset under simulated greenhouse background noise conditions. Interestingly, looking at the raw number of correctly classified *B. terrestris* samples as displayed in Fig 8, the MM model still correctly classifies almost as many samples as the RS model because of the large difference in test dataset size.

## Discussion

Summarising the results, the dataset comparison clearly showed the impressive capabilities of high-end measurement equipment and the shortcomings of low-cost devices used for a task as demanding as acoustic insect sound recording and recognition. Whereas the MM allowed for the recording of a dataset of impressive size, encompassing sounds much below human perception abilities, generating a dataset of sufficient size to train a deep learning sound recognition model turned out to be tedious with the low-cost hardware.

Comparing the different insect classes and their recordings using the two different setups gives some insight into the different insects’ acoustic behaviour. Insects that are loud and active will be picked up by both microphones almost equally capably and often. This can be observed in the numbers of *B. terrestris* and, to a lesser extent, also in *E. balteatus*. Insects that are very active but quiet will be frequently recorded by the MM but not by the RS. Examples of this are *A. aphidimyza* and *B. difformis*.

However, it must be noted that the recording conditions to create these two sound datasets differ vastly from conditions where an insect recognition system might be deployed in practice in terms of the many factors that might influence the different insect activity levels. For instance, one could hypothesise that the relatively small cage used to keep the insects in front of the microphone might suppress flight activity in larger insects more than it does in smaller insects. Further, the absence of external stimuli, such as wind, different plant odours and the absence of other insect species (e.g., prey or predators) within the recording environment might all influence the insect behaviour recorded in this study. Finally, all insects were recorded in different numbers inside the cage, which makes inferring insect activity from recording yield rate rather inaccurate.

Nevertheless, looking at the low end of the yield rate spectrum, it is interesting to note that the three lowest yield rates recorded are of pest insects. This could lead one to the hypothesis that, in general, most pest insects will have less of an incentive to move, and therefore be acoustically active while feeding on a plant than predators, which would need to hunt for prey, or pollinators searching for flowers. However, reviewing the results also presents a few contrary examples, such as the behaviour of the fungus gnat *B. difformis*, which, although a pest insect, was still very active during recordings. In the case of fungus gnats, the imagoes do not feed on plants as their larvae do, but instead spend their short lives solely flying around looking to mate and find a spot for oviposition [35]. In conclusion, while the quantitative analysis of the recordings made in this study provides some interesting starting points to discuss the influences of acoustic insect activity on the prospects of acoustic insect recognition systems, an adequate entomological discussion of the individual insect behaviours is far beyond the scope of this paper.

When analysing the composition of the different datasets regarding the SPL of different samples, it is interesting to note that many of the low-level sounds recorded using the MM did not seem to benefit the recognition models trained in the environmental sound simulation. Rather, these very low-level sounds appeared to be masked quite quickly when approaching real-world sound conditions during the gradual training in the environmental sound simulation. Instead, the great number of low-level sounds that became unrecognisable once background sounds were introduced seemed to destabilise the training process and finally even appeared to hinder the MM model from learning to recognise even the louder and therefore easy-to-classify sound samples in the MM dataset. This effect, already observed in [11], persisted even with the use of the SPL-weighted loss function designed to specifically mitigate it.

However, it must be mentioned here, that to allow for a comparison of the data quality instead of quantity, both models were trained with a comparable number of samples. Therefore, the MM model could not leverage the full potential of the larger MM dataset.

The RS model struggled with exactly the opposite problem. The very sparse number of sound samples for quieter insects prevented the models from learning to recognise these sounds altogether. However, for the louder insects, a noise robust recognition of their loud sounds could be achieved.

Combining the analysis of the SPL of correctly and incorrectly classified sound samples in the case of *B. terrestris* with the SPL distribution in the MM and RS dataset for this class clearly shows that the MM dataset contains many sounds that are too quiet to be classified correctly (< 15 dB), especially in the presence of background noises. The RS dataset on the other hand misses out on a lot of sounds that could very well benefit the function of an insect recognition system (sounds between 15 dB and 38 dB).

Looking at the limitations of this study, it must be mentioned that all results presented here were obtained either under pure laboratory conditions or in simulated greenhouse conditions. In theory, mixing background sound recordings with laboratory insect recordings should provide a good estimation of real-world sounds. The only obvious deviation is the summation of self-noise from both recordings, leading to double the amount of self-noise in the simulated recordings compared to real-world recordings. The simulation results presented here should therefore underestimate real-world performances. However, validation experiments using real-world recordings are ultimately required to validate the estimations presented in this study. Only relying on simulation data regarding the influence of environmental noise must be considered a limitation of this study.

Further, comparing only two microphone arrays is another limitation of this study, and certainly only presents a glimpse into the wide variety of available hardware. While the selected high-end solution seemed to portray the performance of acoustic laboratory equipment well, the low-cost solution selected here – the RS – is only one of many possible low-cost solutions. Besides the MEMS microphones used in the RS, there are electret microphones, which could provide another low-cost microphone option. However, regarding their utilization in microphone arrays as desirable for acoustic insect detection applications [11], a drawback of such electret microphones is that they require individual calibration, whereas MEMS microphones can be manufactured to such tight tolerances that calibration is not necessary.

## Conclusions

By comparing a low- and high-budget microphone array setup for recording and recognising insect sounds, this study was able to show the need for the development of dedicated insect acoustic sensors. While the expensive measurement microphone array showed impressive potential to record very quiet sounds, the majority of these quiet sounds appeared to be of little benefit to recognition systems designed to operate during the presence of normal background noise. Future work should investigate excluding the most quiet sounds from a laboratory dataset when it is being used to train models with applications in real-world noisy conditions. The comparison with the low-budget array did show that, in contrast, the currently available low-cost MEMS microphones miss out on a lot of mid-level sounds that could be beneficial to an insect recognition system.

Because almost all low-cost microphones available today are designed for human speech applications, which require a different frequency range and are less demanding regarding the necessary microphone sensitivity, this study highlights a gap in the market of available hardware. To further advance the field of acoustic insect recognition towards practical usable products with applications in targeted pest and beneficial insect monitoring, as well as general biodiversity monitoring, dedicated acoustic sensor hardware is needed at a reasonable price. Such microphones would ideally provide a combination of sensitivity and self-noise that allows for the recording of insect sounds down to about 15 dB, which seemed to be the lower bound for sounds that were useful to the models in this study. Critically, these microphones should be optimised for recordings in the relevant low-frequency range upwards of 50 Hz where most insect wing beat frequencies (and therefore flight sound base frequency) reside.

Microphone quality aside, an insect’s acoustic activity remains a critical prerequisite for its recognition using any microphone. The discussion around the acoustic activity of the different insects recorded showed that this critical factor, which ultimately determines the prospects of successful acoustic recognition of an insect, does not only correlate with insect size, but rather is highly individual and mainly influenced by its mode of behaviour.

To accelerate the research, future work could explore ways of using data recorded with high-quality microphones in the process of training a model operating on low-cost hardware in a transfer learning manner. Such methods could accelerate the development of acoustic insect sensors by leveraging existing public laboratory sound datasets, such as the recently published InsectSound1000 dataset [12], to improve the performance of a system operating on different, lower-cost hardware.

This leads to the following recommendations:

- Future work developing insect sound recognition models using the InsectSound1000 dataset could explore excluding the very quietest sounds from the training dataset to mitigate their destabilizing effect on model training.
- Future work could further investigate alternative existing low-cost microphones regarding their performance in the low-frequency range.
- Ultimately, future work looking to develop insect sound recognition systems for commercial use in agriculture, horticulture or biodiversity monitoring should explore the development of purpose-built insect recording MEMS microphones and their use in building microphone arrays.
- Future work should seek to validate the environmental sound simulation approach utilised in this study by evaluating the models presented here on insect recordings obtained in real-world greenhouses.
- Finding effective ways to utilise high-quality, multi-channel laboratory recording datasets as pretraining material for sound recognition systems operation on different hardware (lower-quality microphones, different number of channels) could accelerate new developments in this area.

## Supporting information

S1 CAD File CAD-file of the measurement microphone array fixture in stl format. The fixture was printed on a multi-jet fusion 3D printer using PA12 material.
(STL)

S2 CAD File CAD-file of the ReSpeaker Core V2.0 housing top half in stl format. The housing was printed on a multi-jet fusion 3D printer using PA12 material.
(STL)

S3 CAD File CAD-file of the ReSpeaker Core V2.0 housing bottom half in stl format. The housing was printed on a multi-jet fusion 3D printer using PA12 material.
(STL)

S4CAD File CAD-file of the ReSpeaker Core V2.0 housing seal in stl format. The seal was printed on a polyjet 3D printer using silicone rubber G1L (Shore hardness 35 A).
(STL)

S1 Fig Spectrograms of one selected insect sound sample per insect and microphone array. Respective RS and MM samples are not parallel recordings, but subjective selections from the two datasets based on the visual clarity of their spectrograms. The spectrograms were created from the loudest channel of the raw 48 kHz recordings. The spectrograms were computed using the scipy signal spectrogram function. The input signal was windowed with a Tukey window (*alpha* = 0.25). The segment length was set to 1083 samples. The overlap between segments was 135 samples. The Fourier transform was performed using 2048 points.
(PDF)

S1 Table Comparison of the data processing. Table comparing the data processing steps taken in preceding work [11] and the work presented in this study.
(PDF)

S1 Appendix Steps of the training data pipeline. (PDF)

S2 Table Data augmentation applied. Table listing the augmentation methods and their value range as applied to the training data during model training.
(PDF)

S3 Table Base and maximum learning rates used. Table listing the learning rates used as base and maximum setting for the cyclical learning rate scheme used. Values where chosen based on a learning rate test performed before every stage of training in the environmental noise simulation.
(PDF)

S2 Appendix Sound Pressure Level Calculation for the Measurement Microphone Array. (PDF)

S3 Appendix Calculation of the Value Range Adjustment Factor for the ReSpeaker Core v2.0 Signals. (PDF)

S2 Fig Three figures showing bar plots of precision, recall and f1-score calculated for every class for the best attempts of training an MM and RS model in the environmental sound simulation. (PDF)

## References

[1] Montgomery GA, Belitz MW, Guralnick RP, Tingley MW. Standards and Best Practices for Monitoring and Benchmarking Insects. Front Ecol Evol. 2021;8. doi: 10.3389/fevo.2020.579193

[2] Bhairavi KS, Bhattacharyya B, Manpoong NS, Das PPG, Devi EB, Bhagawati S. Recent advances in exploration of acoustic pest management: A review. Journal of Entomology and Zoology Studies. 2020;8:2056–61.

[3] Cardim Ferreira Lima M, Damascena de Almeida Leandro ME, Valero C, Pereira Coronel LC, Gonçalves Bazzo CO. Automatic Detection and Monitoring of Insect Pests—A Review. Agriculture. 2020;10(5):161. doi: 10.3390/agriculture10050161

[4] Gibb R, Browning E, Glover‐Kapfer P, Jones KE. Emerging opportunities and challenges for passive acoustics in ecological assessment and monitoring. Methods Ecol Evol. 2018;10(2):169–85. doi: 10.1111/2041-210x.13101

[5] Müller-Blenkle C, Simon U, Meyer R, Szallies I, Lorenz D, Prozell S. A method for acoustic storage pest detection and its challenges. Journal of Cultivated Plants. 2023;75(09–10):235–47. doi: 10.5073/JFK.2023.09-10.02

[6] Souza UBd, Escola JPL, Maccagnan DHB, Brito L da C, Guido RC. Empirical mode decomposition applied to acoustic detection of a cicadid pest. Computers and Electronics in Agriculture. 2022;199:107181. doi: 10.1016/j.compag.2022.107181

[7] Li Y, Kiskin I, Sinka M, Zilli D, Chan H, Herreros-Moya E. Fast mosquito acoustic detection with field cup recordings: an initial investigation. In: 2018. https://api.semanticscholar.org/CorpusID:245426611

[8] Noda JJ, Travieso-González CM, Sánchez-Rodríguez D, Alonso-Hernández JB. Acoustic classification of singing insects based on MFCC/LFCC fusion. Applied Sciences. 2019;9(19):4097. doi: 10.3390/app9194097

[9] Branding J, von Hörsten D, Wegener JK. Akustische Insektenerkennung – Deep Learning zur Klassifikation leisester Fluggeräusche: Acoustic insect detection – deep learning for classification of low level flight sounds. In: Lecture Notes in Informatics (LNI) - Proceedings, 2022. https://dl.gi.de/handle/20.500.12116/38433

[10] Chhetri A, Hilmes P, Kristjansson T, Chu W, Mansour M, Li X. Multichannel Audio Front-End for Far-Field Automatic Speech Recognition. In: 2018. 1527–31. 10.23919/EUSIPCO.2018.8553149

[11] Branding J, von Hörsten D, Wegener JK, Böckmann E, Hartung E. Towards noise robust acoustic insect detection: from the lab to the greenhouse. KI - Künstliche Intelligenz. 2023. doi: 10.1007/s13218-023-00812-x

[12] Branding J, von Hörsten D, Böckmann E, Wegener JK, Hartung E. InsectSound1000 An insect sound dataset for deep learning based acoustic insect recognition. Sci Data. 2024;11(1):475. doi: 10.1038/s41597-024-03301-4 38724595 PMC11082239

[13] Kjaer B. Product data: ½” low-noise free-field teds microphones type 4955 and 4955-a. https://www.bksv.com/-/media/literature/Product-Data/bp2148.ashx 2024 August 27.

[14] Electronics K. Product data sheet SPU0414HR5H-SB. https://www.mouser.de/datasheet/2/218/knowles_01232019_SPU0414HR5H_SB-1891952.pdf 2024 August 27.

[15] Seeed Studio I. ReSpeaker Core v2.0. https://wiki.seeedstudio.com/ReSpeaker_Core_v2.0/ 2026 January 28.

[16] Branding J, von Hörsten D, Böckmann E, Wegener JK, Hartung E. InsectSound1000-MEMS. OpenAgrar Repository. 2026. 10.5073/20260401-141040-0

[17] Branding J, von Hörsten D, Böckmann E, Wegener JK, Hartung E. InsectSound1000-GreenhouseNoiseRecordings. OpenAgrar Repository. 2026. 10.5073/20260415-170032-0

[18] Hoshen Y, Weiss RJ, Wilson KW. Speech acoustic modeling from raw multichannel waveforms. In: 2015 IEEE International Conference on Acoustics, Speech and Signal Processing (ICASSP), 2015. 4624–8. 10.1109/icassp.2015.7178847

[19] McCowan I. Microphone Arrays: A Tutorial. http://www.aplu.ch/home/download/microphone_array.pdf 2001.

[20] van den Oord A, Dieleman S, Zen H, Simonyan K, Vinyals O, Graves A. WaveNet: A Generative Model for Raw Audio. 2016. http://arxiv.org/pdf/1609.03499v2

[21] Pandey SK, Shekhawat HS, Prasanna SRM. Emotion Recognition from Raw Speech using Wavenet. In: TENCON 2019 - 2019 IEEE Region 10 Conference (TENCON), 2019. 1292–7. 10.1109/tencon.2019.8929257

[22] Oh SL, Jahmunah V, Ooi CP, Tan R-S, Ciaccio EJ, Yamakawa T, et al. Classification of heart sound signals using a novel deep WaveNet model. Comput Methods Programs Biomed. 2020;196:105604. doi: 10.1016/j.cmpb.2020.105604 32593061

[23] Zhang X, Gao Y, Yu Y, Li W. Music Artist Classification with WaveNet Classifier for Raw Waveform Audio Data;. http://arxiv.org/pdf/2004.04371v1

[24] Sainath TN, Weiss RJ, Wilson KW, Narayanan A, Bacchiani M. In: 2016. 5075–9. doi: 10.1109/ICASSP.2016.7472644

[25] Branding J. NBF-WaveNet. https://github.com/Jelt0/NBF-WaveNet 2024.

[26] Hochreiter S, Bengio Y, Frasconi P, Schmidhuber J. Gradient flow in recurrent nets: the difficulty of learning long-term dependencies. A Field Guide to Dynamical Recurrent Neural Networks. IEEE Press. 2001.

[27] Ozer I, Ozer Z, Findik O. Noise robust sound event classification with convolutional neural network. Neurocomputing. 2018;272:505–12. doi: 10.1016/j.neucom.2017.07.021

[28] Abadi M, Agarwal A, Barham P, Brevdo E, Chen Z, Citro C. TensorFlow: Large-Scale Machine Learning on Heterogeneous Distributed Systems. 2015. http://download.tensorflow.org/paper/whitepaper2015.pdf

[29] Kingma DP, Ba J. Adam: A Method for Stochastic Optimization. 2014. http://arxiv.org/pdf/1412.6980v9

[30] Wei S, Zou S, Liao F, Lang W. A Comparison on Data Augmentation Methods Based on Deep Learning for Audio Classification. J Phys: Conf Ser. 2020;1453(1):012085. doi: 10.1088/1742-6596/1453/1/012085

[31] Smith LN. Cyclical Learning Rates for Training Neural Networks. http://arxiv.org/pdf/1506.01186v6 2015.

[32] Le-Qing Z. Insect Sound Recognition Based on MFCC and PNN. In: 2011 International Conference on Multimedia and Signal Processing, 2011. 42–6. 10.1109/cmsp.2011.100

[33] Priyadarshani N, Marsland S, Castro I. Automated birdsong recognition in complex acoustic environments: a review. Journal of Avian Biology. 2018;49(5). doi: 10.1111/jav.01447

[34] Astapov S, Lavrentyev A, Shuranov E. Far Field Speech Enhancement at Low SNR in Presence of Nonstationary Noise Based on Spectral Masking and MVDR Beamforming. Lecture Notes in Computer Science. Springer International Publishing. 2018. p. 21–31. 10.1007/978-3-319-99579-3_3

[35] Harris MA, Gardner WA, Oetting RD. A review of the scientific literature on fungus gnats (Diptera: Sciaridae) in the genus Bradysia. Journal of Entomological Science. 1996;31(3):252–76. doi: 10.18474/0749-8004-31.3.252

