# Supplementary material for: Why we need dedicated insect microphones - A comparison between measurement and MEMS microphone arrays highlights gap in available hardware
Source: PLoS One. 2026 Jul 8;21(7):e0350946. doi: 10.1371/journal.pone.0350946 (PMC13345237; doi:10.1371/journal.pone.0350946)
Supplement: S1 Fig — Respective RS and MM samples are not parallel recordings, but subjective selections from the two datasets based on the visual clarity of their spectrograms. The spectrograms were created from the loudest channel of the raw 48 kHz recordings. The spectrograms were computed using the scipy signal spectrogram function. The input signal was windowed with a Tukey window (alpha = 0.25). The segment length was set to 1083 samples. The overlap between segments was 135 samples. The Fourier transform was performed using 2048 points. (PDF) [file pone.0350946.s005.pdf]

Supporting Information for:

Why we need dedicated insect microphones
A comparison between measurement and MEMS microphone arrays highlights gap in available hardware

Jelto Branding[1¤*], Dieter von Hörsten[1], Elias Böckmann[2], Jens Karl Wegener[1], Eberhard Hartung[3],

1 Julius Kühn Institute (JKI), Institute for Application Techniques in Plant Protection, Messeweg 11/12, 38104 Braunschweig, Germany
2 Julius Kühn Institute (JKI), Institute for Plant Protection in Horticulture and Urban Green, Messeweg 11/12, 38104 Braunschweig, Germany
3 Christian-Albrechts-Universität zu Kiel, Institute of Agricultural Process Engineering, Max-Eyth-Str. 6, 24118 Kiel, Germany

¤Current Address: Christian-Albrechts-Universität zu Kiel, Institute of Agricultural Process Engineering, Max-Eyth-Str. 6, 24118 Kiel, Germany
* jbranding@ilv.uni-kiel.de

S1 Fig.

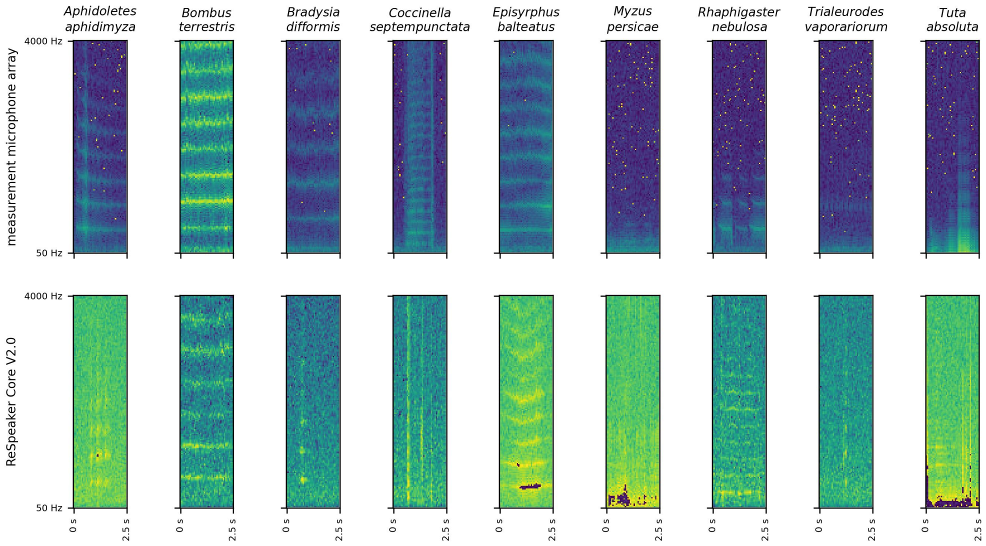

Fig 1. Spectrograms of one selected insect sound sample per insect and microphone array. Respective ReSpeaker Core V2.0 (RS) and measurement microphone array (MM) samples are not parallel recordings, but subjective selections from the two datasets based on the visual clarity of their spectrograms. The spectrograms were created from the loudest channel of the raw 48 kHz recordings. The spectrograms were computed using the scipy signal spectrogram function. The input signal was windowed with a Tukey window ($alpha = 0.25$). The segment length was set to 1083 samples. The overlap between segments was 135 samples. The Fourier transform was performed using 2048 points.
