# Supplementary material for: Why we need dedicated insect microphones - A comparison between measurement and MEMS microphone arrays highlights gap in available hardware
Source: PLoS One. 2026 Jul 8;21(7):e0350946. doi: 10.1371/journal.pone.0350946 (PMC13345237; doi:10.1371/journal.pone.0350946)
Supplement: S1 Table — Table comparing the data processing steps taken in preceding work [11] and the work presented in this study. (PDF) [file pone.0350946.s006.pdf]

Supporting Information for:

Why we need dedicated insect microphones

A comparison between measurement and MEMS microphone arrays highlights gap in available hardware

Jelto Branding[1¤*], Dieter von Hörsten[1], Elias Böckmann[2], Jens Karl Wegener[1], Eberhard Hartung[3],

1 Julius Kühn Institute (JKI), Institute for Application Techniques in Plant Protection, Messeweg 11/12, 38104 Braunschweig, Germany
2 Julius Kühn Institute (JKI), Institute for Plant Protection in Horticulture and Urban Green, Messeweg 11/12, 38104 Braunschweig, Germany
3 Christian-Albrechts-Universität zu Kiel, Institute of Agricultural Process Engineering, Max-Eyth-Str. 6, 24118 Kiel, Germany

¤Current Address: Christian-Albrechts-Universität zu Kiel, Institute of Agricultural Process Engineering, Max-Eyth-Str. 6, 24118 Kiel, Germany
* jbranding@ilv.uni-kiel.de

S1 Table

	preceding work	this study
**Data**	measurement microphone array (MM) insect recordings of 5 insects and background sound recordings with MM	MM recordings of 9 insects + ReSpeaker Core V2.0 (RS) recordings and background sound recordings with MM and RS
**Insect dataset preparation**	sound sample extraction for MM data as described in [1]	sound sample extraction for MM data as described in [1] and sound sample extraction for RS data based on [1] with two adjusted parameters (see section Sound Sample Extraction in the main article)
**Insect dataset split**	classic 60-20-20-training-validation-test split	split aiming for a 60-20-20 distribution, but considering separating recording dates between the splits.
**Model**	NBF-WaveNet 4 channel input (for MM data) and 5 classes output	NBF-WaveNet 4 channel input (for MM) and 9 classes output and NBF-WaveNet 6 channel input (for RS) and 9 classes output
**Loss function**	standard: categorical cross-entropy loss	modified: SPL-weighted loss
**Learning rate selection**	grid search	learning rate tests based on [2]
**Learning rate behaviour**	constant	cyclical learning rate scheme based on [2]
**Environmental noise level steps [%]**	0 - 10 - 20 - 100	0 - 1 - 10 - 100

Table 1. Comparison of the data processing. Table comparing the data processing steps taken in preceding work [3] and the work presented in this study.

References

1. Branding J, von Hörsten D, Wegener JK, Böckmann E, Hartung E. InsectSound1000 An insect sound dataset for deep learning based acoustic insect recognition. Scientific Data. 2024 May;11. doi:10.1038/s41597-024-03301-4.

2. Smith LN. Cyclical Learning Rates for Training Neural Networks; 2015. Available from: `http://arxiv.org/pdf/1506.01186v6`.

3. Branding J, von Hörsten D, Wegener JK, Böckmann E, Hartung E. Towards noise robust acoustic insect detection: from the lab to the greenhouse. KI - Künstliche Intelligenz. 2023. doi:10.1007/s13218-023-00812-x.
