# Supplementary material for: Why we need dedicated insect microphones - A comparison between measurement and MEMS microphone arrays highlights gap in available hardware
Source: PLoS One. 2026 Jul 8;21(7):e0350946. doi: 10.1371/journal.pone.0350946 (PMC13345237; doi:10.1371/journal.pone.0350946)
Supplement: S1 Appendix — (PDF) [file pone.0350946.s007.pdf]

Supporting Information for:

Why we need dedicated insect microphones
A comparison between measurement and MEMS microphone arrays highlights gap in available hardware

Jelto Branding[1¤*], Dieter von Hörsten[1], Elias Böckmann[2], Jens Karl Wegener[1], Eberhard Hartung[3],

1 Julius Kühn Institute (JKI), Institute for Application Techniques in Plant Protection, Messeweg 11/12, 38104 Braunschweig, Germany
2 Julius Kühn Institute (JKI), Institute for Plant Protection in Horticulture and Urban Green, Messeweg 11/12, 38104 Braunschweig, Germany
3 Christian-Albrechts-Universität zu Kiel, Institute of Agricultural Process Engineering, Max-Eyth-Str. 6, 24118 Kiel, Germany

¤Current Address: Christian-Albrechts-Universität zu Kiel, Institute of Agricultural Process Engineering, Max-Eyth-Str. 6, 24118 Kiel, Germany
* jbranding@ilv.uni-kiel.de

S1 Appendix Steps of the training data pipeline.
The training data pipeline feeding the datasets stored on disc to the model consisted of the following steps:

1. Building lists of training and validation insect sound files for every class, either from measurement microphone array (MM) or ReSpeaker Core V2.0 (RS) data.
2. Balancing training data files by over- and undersampling the data for every label to a target number of training samples.
3. Shuffling insect training data files (and reshuffling every epoch of training).
4. Applying data augmentation to the training files.

In order to be able to mix insect and environmental sounds for implementation reasons, the same number of both sound files was needed. As the dataset offers far fewer different environmental sound samples than insect sounds, the following steps were necessary:

5. Over- or undersampling the lists of available training and validation environmental sound files to match the total number of training or validation insect sounds.
6. Mixing the environmental sounds with the insect training and validation sounds.
7. Only when processing MM data: clip the signals to a value range of -10 to 10, mimicking the behaviour of the A/D converter. The large dynamic range of the RS means it will practically never have issues with clipping the signal with the data encountered in this study.
8. Prefiltering the signal by:
   - 8.1 Applying a fourth-order high-pass Butterworth filter at 50 Hz.
   - 8.2 Converting the data to a value range of -1 to 1. This is done by dividing the MM data by 10 and by multiplying the RS data by the correction factor a, deduced in section SPL Calculation in the main article, divided by 10.
   - 8.3 Centring the signal around 0 by element-wise subtracting the signal mean.
