# Supplementary material for: Why we need dedicated insect microphones - A comparison between measurement and MEMS microphone arrays highlights gap in available hardware
Source: PLoS One. 2026 Jul 8;21(7):e0350946. doi: 10.1371/journal.pone.0350946 (PMC13345237; doi:10.1371/journal.pone.0350946)
Supplement: S2 Table — Table listing the augmentation methods and their value range as applied to the training data during model training. (PDF) [file pone.0350946.s008.pdf]

Supporting Information for:

Why we need dedicated insect microphones
A comparison between measurement and MEMS microphone arrays highlights gap in available hardware

Jelto Branding[1¤*], Dieter von Hörsten[1], Elias Böckmann[2], Jens Karl Wegener[1], Eberhard Hartung[3],

1 Julius Kühn Institute (JKI), Institute for Application Techniques in Plant Protection, Messeweg 11/12, 38104 Braunschweig, Germany
2 Julius Kühn Institute (JKI), Institute for Plant Protection in Horticulture and Urban Green, Messeweg 11/12, 38104 Braunschweig, Germany
3 Christian-Albrechts-Universität zu Kiel, Institute of Agricultural Process Engineering, Max-Eyth-Str. 6, 24118 Kiel, Germany

¤Current Address: Christian-Albrechts-Universität zu Kiel, Institute of Agricultural Process Engineering, Max-Eyth-Str. 6, 24118 Kiel, Germany
* jbranding@ilv.uni-kiel.de

S2 Table

Augmentation method	Value range
time shift	±250 ms
random gain	±20 %
signal inversion	Yes or No

Table 1. Data augmentation applied. Table listing the augmentation methods and their value range as applied to the training data during model training.
