# Supplementary material for: Why we need dedicated insect microphones - A comparison between measurement and MEMS microphone arrays highlights gap in available hardware
Source: PLoS One. 2026 Jul 8;21(7):e0350946. doi: 10.1371/journal.pone.0350946 (PMC13345237; doi:10.1371/journal.pone.0350946)
Supplement: S3 Table — Table listing the learning rates used as base and maximum setting for the cyclical learning rate scheme used. Values where chosen based on a learning rate test performed before every stage of training in the environmental noise simulation. (PDF) [file pone.0350946.s009.pdf]

Supporting Information for:

Why we need dedicated insect microphones

A comparison between measurement and MEMS microphone arrays highlights gap in available hardware

Jelto Branding[1¤*], Dieter von Hörsten[1], Elias Böckmann[2], Jens Karl Wegener[1], Eberhard Hartung[3],

1 Julius Kühn Institute (JKI), Institute for Application Techniques in Plant Protection, Messeweg 11/12, 38104 Braunschweig, Germany
2 Julius Kühn Institute (JKI), Institute for Plant Protection in Horticulture and Urban Green, Messeweg 11/12, 38104 Braunschweig, Germany
3 Christian-Albrechts-Universität zu Kiel, Institute of Agricultural Process Engineering, Max-Eyth-Str. 6, 24118 Kiel, Germany

¤Current Address: Christian-Albrechts-Universität zu Kiel, Institute of Agricultural Process Engineering, Max-Eyth-Str. 6, 24118 Kiel, Germany
* jbranding@ilv.uni-kiel.de

S3 Table

Noise amplitude [%]	ReSpeaker		Measurement Microphone Array	
	**Base LR**	**Max. LR**	**Base LR**	**Max. LR**
0	1.50e-04	5.00e-04	2.00e-04	5.00e-04
1	1.50e-05	5.00e-04	1.50e-05	5.00e-04
10	4.00e-05	5.00e-04	1.50e-05	5.00e-04
100	4.00e-05	5.00e-04	1.50e-05	5.00e-04

Table 1. Base and maximum learning rates used. Table listing the learning rates used as base and maximum setting for the cyclical learning rate scheme used. Values were chosen based on a learning rate test performed before every stage of training in the environmental noise simulation.
