# Supplementary material for: Why we need dedicated insect microphones - A comparison between measurement and MEMS microphone arrays highlights gap in available hardware
Source: PLoS One. 2026 Jul 8;21(7):e0350946. doi: 10.1371/journal.pone.0350946 (PMC13345237; doi:10.1371/journal.pone.0350946)
Supplement: S2 Appendix — (PDF) [file pone.0350946.s010.pdf]

Supporting Information for:

Why we need dedicated insect microphones

A comparison between measurement and MEMS microphone arrays highlights gap in available hardware

Jelto Branding[1¤*], Dieter von Hörsten[1], Elias Böckmann[2], Jens Karl Wegener[1], Eberhard Hartung[3],

1 Julius Kühn Institute (JKI), Institute for Application Techniques in Plant Protection, Messeweg 11/12, 38104 Braunschweig, Germany
2 Julius Kühn Institute (JKI), Institute for Plant Protection in Horticulture and Urban Green, Messeweg 11/12, 38104 Braunschweig, Germany
3 Christian-Albrechts-Universität zu Kiel, Institute of Agricultural Process Engineering, Max-Eyth-Str. 6, 24118 Kiel, Germany

¤Current Address: Christian-Albrechts-Universität zu Kiel, Institute of Agricultural Process Engineering, Max-Eyth-Str. 6, 24118 Kiel, Germany
* jbranding@ilv.uni-kiel.de

S2 Appendix Sound Pressure Level Calculation for the Measurement Microphone Array

To calculate the sound pressure level (SPL) of the measurement microphone array (MM) samples, the same preprocessing steps that were applied to the signals before feeding them into the model were applied. Namely, a fourth-order high-pass Butterworth filter at 50 Hz was applied, and the signals were centred around zero by subtracting the signal mean. The different microphone sensitivities of the four microphones were accounted for by dividing each of the four recorded channels by the corresponding microphone sensitivity according to the following equation 1:

$$y_{MM_2} = \frac{y_{MM_1}[\mathrm{V}]}{sensitivity[\mathrm{V\,Pa^{-1}}]} \tag{1}$$

The signals were then corrected for the analogue gain applied to the signal from the different devices in the measurement chain according to equation 2. In this case, the Brüel & Kjaer Nexus was set up to amplify each microphone channel with a gain of $g = 20\,\mathrm{dB}$.

$$y_{MM_3}[\mathrm{Pa}] = y_{MM_2} \times 10^{\frac{g[\mathrm{dB}]}{20}} \tag{2}$$

Following this, the SPL in dB was calculated from the multichannel signal by first calculating the root mean square (RMS) value of each channel, following equation 3, and then converting the mean value of all channels from Pa to dB according to equation 4.

$$rms_{MM} = \sqrt{mean(y_{MM_3}^2)} \tag{3}$$

$$SPL_{MM}[\mathrm{dB}] = 20 * \log_{10} \frac{mean(rms_{MM})}{2 \times 10^{-5}\,\mathrm{Pa}} \tag{4}$$
