# Supplementary material for: Why we need dedicated insect microphones - A comparison between measurement and MEMS microphone arrays highlights gap in available hardware
Source: PLoS One. 2026 Jul 8;21(7):e0350946. doi: 10.1371/journal.pone.0350946 (PMC13345237; doi:10.1371/journal.pone.0350946)
Supplement: S3 Appendix — (PDF) [file pone.0350946.s011.pdf]

# Supporting Information for:

## Why we need dedicated insect microphones
A comparison between measurement and MEMS microphone arrays highlights gap in available hardware

Jelto Branding[1¤*], Dieter von Hörsten[1], Elias Böckmann[2], Jens Karl Wegener[1], Eberhard Hartung[3],

**1** Julius Kühn Institute (JKI), Institute for Application Techniques in Plant Protection, Messeweg 11/12, 38104 Braunschweig, Germany
**2** Julius Kühn Institute (JKI), Institute for Plant Protection in Horticulture and Urban Green, Messeweg 11/12, 38104 Braunschweig, Germany
**3** Christian-Albrechts-Universität zu Kiel, Institute of Agricultural Process Engineering, Max-Eyth-Str. 6, 24118 Kiel, Germany

¤Current Address: Christian-Albrechts-Universität zu Kiel, Institute of Agricultural Process Engineering, Max-Eyth-Str. 6, 24118 Kiel, Germany
* jbranding@ilv.uni-kiel.de

### S3 Appendix Calculation of the Value Range Adjustment Factor for the ReSpeaker Core v2.0 Signals

To calculate the sound pressure level (SPL)-like values for the ReSpeaker Core V2.0 (RS) sound samples, the simultaneous background sound recordings made with both microphone arrays in the greenhouse were used to estimate an adjustment factor. As almost all sounds in the background sound dataset are far-field sounds, the sounds recorded by the two microphone arrays placed close to each other in the greenhouse were assumed to be identical.

In detail, both the RS and measurement microphone array (MM) recordings from the greenhouse were manually aligned and cut into exactly simultaneous clips. These clips were then processed using the same steps as described in S1 Appendix, including downsampling to a sampling rate of 16 kHz, applying a high-pass filter at 50 Hz and an offset correction. Following this, the adjustment factor $a$ was calculated as given in equation 1 below:

$$a = \frac{rms_{MM_greenhouse}}{\sqrt{mean(y^2_{RS_greenhouse})}} \tag{1}$$
