# Supplementary material for: Why we need dedicated insect microphones - A comparison between measurement and MEMS microphone arrays highlights gap in available hardware
Source: PLoS One. 2026 Jul 8;21(7):e0350946. doi: 10.1371/journal.pone.0350946 (PMC13345237; doi:10.1371/journal.pone.0350946)
Supplement: S2 Fig — (PDF) [file pone.0350946.s012.pdf]

Supporting Information for:

Why we need dedicated insect microphones

A comparison between measurement and MEMS microphone arrays highlights gap in available hardware

Jelto Branding[1¤*], Dieter von Hörsten[1], Elias Böckmann[2], Jens Karl Wegener[1], Eberhard Hartung[3],

1 Julius Kühn Institute (JKI), Institute for Application Techniques in Plant Protection, Messeweg 11/12, 38104 Braunschweig, Germany
2 Julius Kühn Institute (JKI), Institute for Plant Protection in Horticulture and Urban Green, Messeweg 11/12, 38104 Braunschweig, Germany
3 Christian-Albrechts-Universität zu Kiel, Institute of Agricultural Process Engineering, Max-Eyth-Str. 6, 24118 Kiel, Germany

¤Current Address: Christian-Albrechts-Universität zu Kiel, Institute of Agricultural Process Engineering, Max-Eyth-Str. 6, 24118 Kiel, Germany
* jbranding@ilv.uni-kiel.de

S2 Fig.

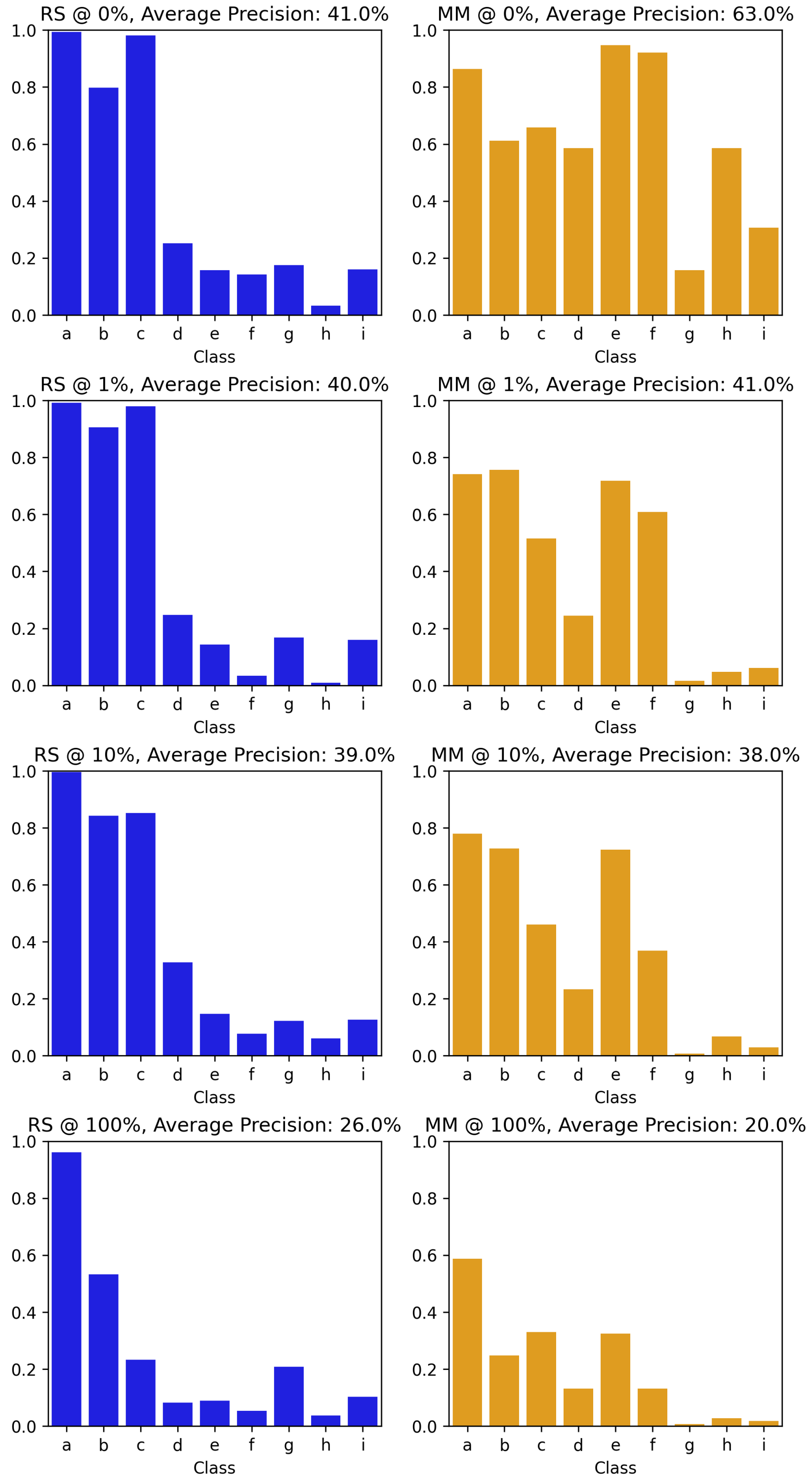

Fig 1. Bar plots showing the precision calculated for every class for the best attempts of training an measurement microphone array (MM) and ReSpeaker Core V2.0 (RS) model in the environmental sound simulation. The macro average precision over all classes is given in each plot title. Classes denoted as: a *Bombus terrestris*, b *Episyrphus balteatus*, c *Rhaphigaster nebulosa*, d *Coccinella septempunctata*, e *Aphidoletes aphidimyza*, f *Bradysia difformis*, g *Tuta absoluta*, h *Myzus persicae*, i *Trialeurodes vaporariorum*, in order of subjective classification difficulty.

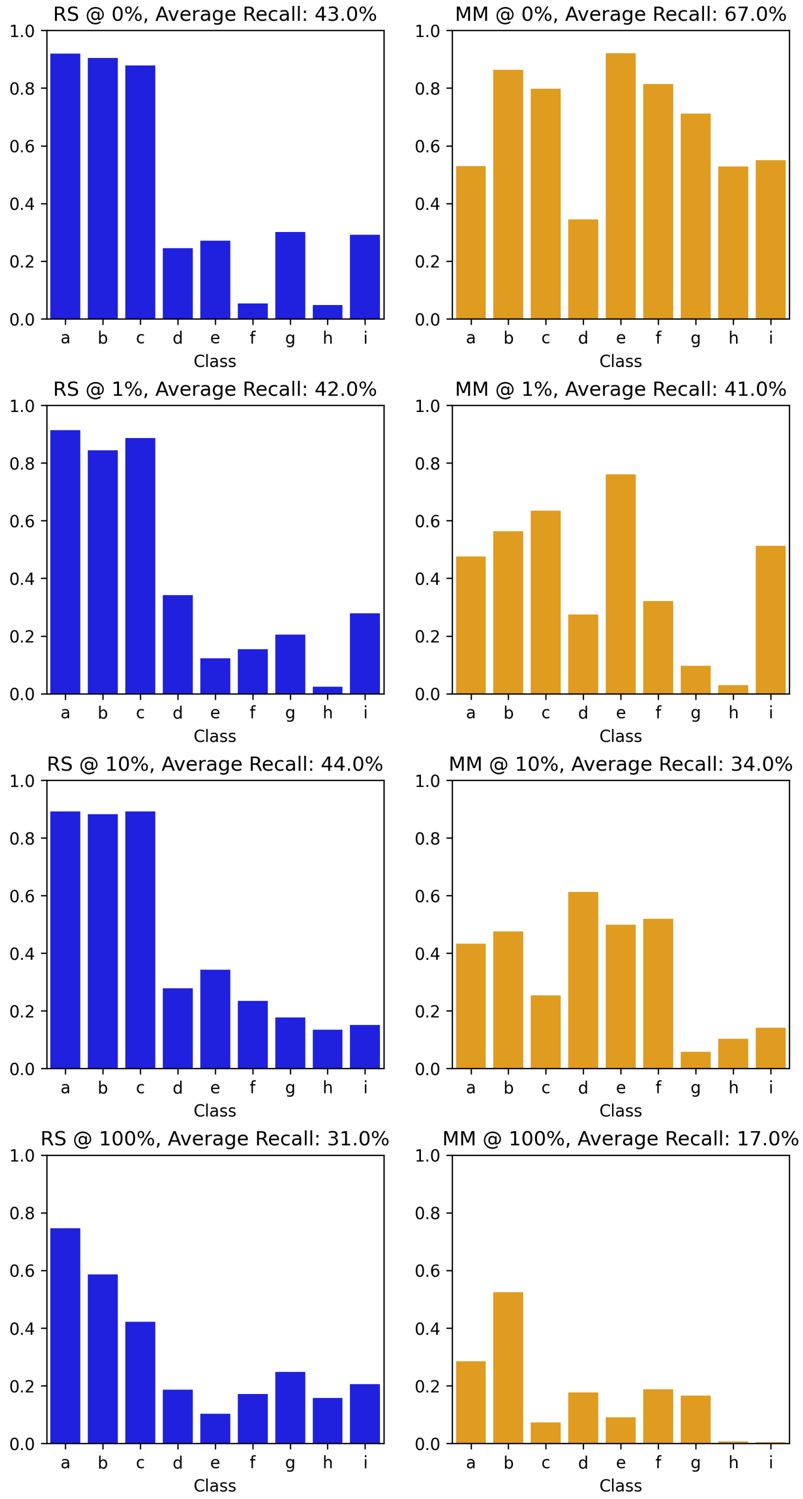

Fig 2. Bar plots showing the recall calculated for every class for the best attempts of training an MM and RS model in the environmental sound simulation. The macro average recall over all classes is given in each plot title. Classes denoted as: a *Bombus terrestris*, b *Episyrphus balteatus*, c *Rhaphigaster nebulosa*, d *Coccinella septempunctata*, e *Aphidoletes aphidimyza*, f *Bradysia difformis*, g *Tuta absoluta*, h *Myzus persicae*, i *Trialeurodes vaporariorum*, in order of subjective classification difficulty.

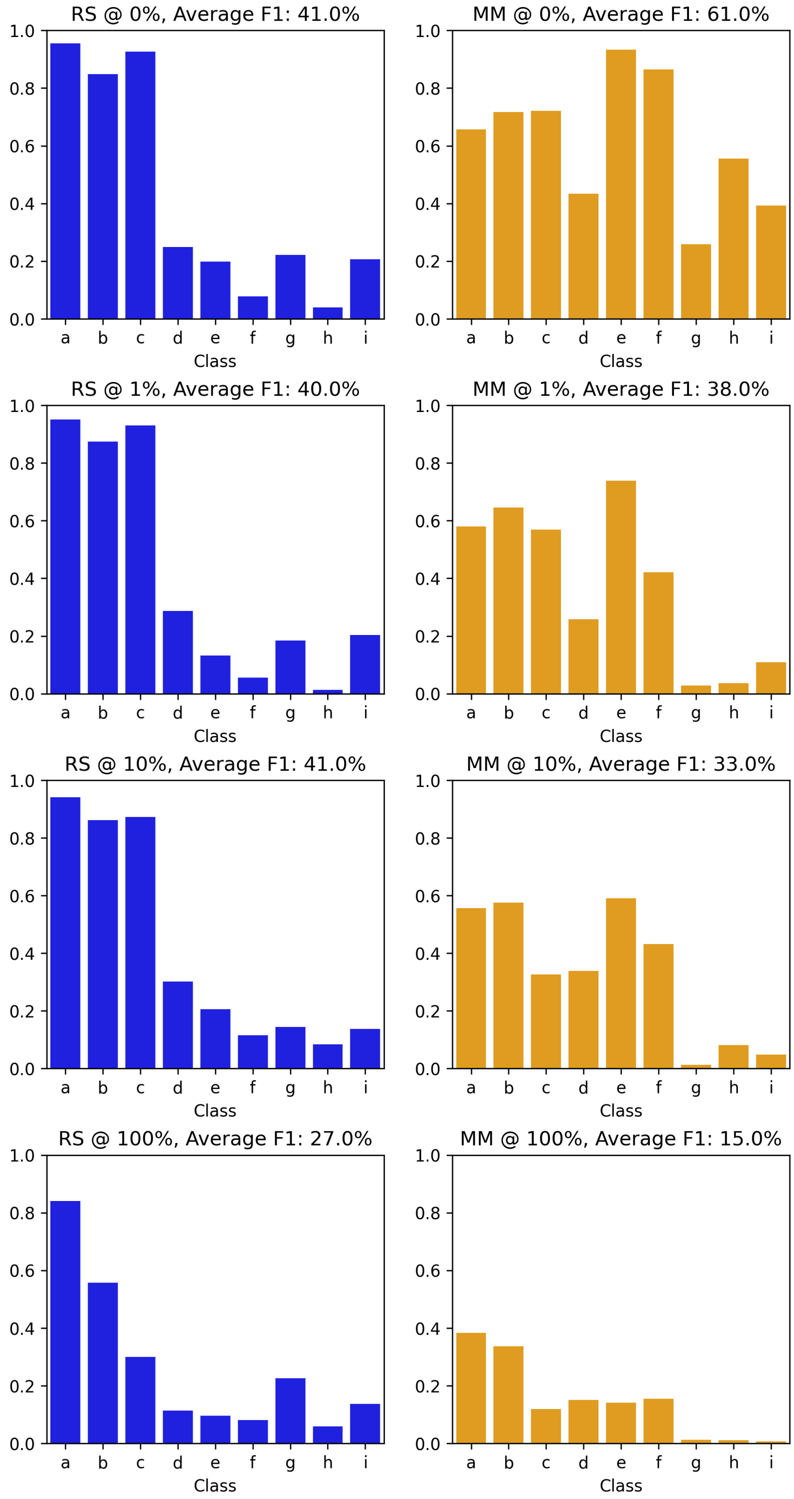

Fig 3. Bar plots showing the f1-score calculated for every class for the best attempts of training an MM and RS model in the environmental sound simulation. The macro average f1-score over all classes is given in each plot title. Classes denoted as: a *Bombus terrestris*, b *Episyrphus balteatus*, c *Rhaphigaster nebulosa*, d *Coccinella septempunctata*, e *Aphidoletes aphidimyza*, f *Bradysia difformis*, g *Tuta absoluta*, h *Myzus persicae*, i *Trialeurodes vaporariorum*, in order of subjective classification difficulty.
